# DEVEA: an interactive shiny application for Differential Expression analysis, data Visualization and Enrichment Analysis of transcriptomics data

**DOI:** 10.12688/f1000research.122949.1

**Published:** 2022-06-28

**Authors:** Miriam Riquelme-Perez, Fernando Perez-Sanz, Jean-François Deleuze, Carole Escartin, Eric Bonnet, Solène Brohard

**Affiliations:** 1 Université Paris-Saclay, CEA, CNRS, MIRCen, Laboratoire des Maladies Neurodégénératives, Fontenay-aux-Roses, 92265, France; 2 Centre National de Recherche en Génomique Humaine (CNRGH), Institut de Biologie François Jacob, CEA, Université Paris-Saclay, Evry, 91000, Evry, France; 3 Biomedical Informatics \& Bioinformatics Service, Institute for Biomedical Research of Murcia (IMIB), Murcia, 30120, Spain

**Keywords:** Bioinformatics, transcriptomics, RNA sequencing, differential expression analysis, enrichment analysis, visualization, R, Shiny, interactive reports.

## Abstract

We are at a time of considerable growth in the use and development of transcriptomics studies and subsequent *in silico* analysis. RNA sequencing is one of the most widely used approaches, now integrated in many studies.

The processing of these data may typically require a noteworthy number of steps, statistical knowledge, and coding skills which is not accessible to all scientists. Despite the undeniable development of software applications over the years to address this concern, it is still possible to improve.

Here we present DEVEA, an R shiny application tool developed to perform differential expression analysis, data visualization and enrichment pathway analysis mainly from transcriptomics data, but also from simpler gene lists with or without statistical values.

Its intuitive and easy-to-manipulate interface facilitates gene expression exploration through numerous interactive figures and tables, statistical comparisons of expression profile levels between groups and further meta-analysis such as enrichment analysis, without bioinformatics expertise.

DEVEA performs a thorough analysis from multiple and flexible input data representing distinct analysis stages. From them, it produces dynamic graphs and tables, to explore the expression levels and statistical differential expression analysis results. Moreover, it generates a comprehensive pathway analysis to extend biological insights. Finally, a complete and customizable HTML report can be extracted for further result exploration outside the application. DEVEA is accessible at https://shiny.imib.es/devea/ and the source code is available on our GitHub repository https://github.com/MiriamRiquelmeP/DEVEA.

## Introduction

RNA sequencing (RNA-seq) has become a routine and popular technique for genome-wide and transcriptomics expression analysis. ^1^ As a result, RNA based measurements and data are extensively incorporated in basic science research and are even increasingly used as molecular diagnostics for human health. These may include diagnosis, prognosis and therapeutic selection. ^2^

However, in order to leverage the full power of this technique, several stages and tools are necessary to translate expression profiles into valuable outcomes. The R statistical environment ^3^ provides many well-known packages to perform key steps of a complete RNA-seq analyses pipeline. Possible examples include differential expression analysis (DEA) functions, leading to lists of differentially expressed genes (DEGs), and annotation enrichment analysis (EA, sometimes called pathway analysis) libraries, which will identify biological pathways or cellular functions significantly enriched from the list of DEGs. Nevertheless, most of these powerful packages are command-line based or demand coding knowledge and are therefore out of reach for scientists with limited computational training. Besides, analyses can be started at different points in the workflow, from raw or partially analysed data from different tools, to individual lists of favourite final features. This is often a limiting factor for the use of rigid tools that import only a single data type. Hence, providing flexible user-friendly tools for the analysis and visualization of gene expression data can help researchers to convert high-throughput genomics into basic science research insights. To bridge this gap, an increasing number of software tools are being released, based on intuitive, point-and-click, graphical interfaces. Frameworks such as R Shiny, ^4^ an R package, facilitate the creation and release of interactive web tools. Some RNA-seq analysis applications from the literature may include iDEP, ^5^ GENAVi ^6^ and ideal, ^7^ among others.

Despite the great progress to facilitate transcriptomics computational analyses, some improvements are still possible for these tools. To this end, we have developed DEVEA, a new interactive R Shiny application for differential expression analysis, data exploration, data visualization and functional enrichment analysis. DEVEA provides an easy-to-use interface to load data in various formats and complexity according to the stage of the analysis (including raw RNA-seq count data, pre-analyzed data, simple lists of genes or proteins obtained from any source or technique, with or without statistical values associated). From them, it generates a wide set of dynamic plots and tables allowing quick navigation through the gene expression profile or enrichment analysis results. The outputs can be downloaded easily and the user can also create custom and operable reports in HTML format. DEVEA is implemented as a publicly available web server and can also be downloaded to be used locally. DEVEA aims to conduct a proper analysis by reaching out both life scientists (gathering the biological expertise) and bioinformaticians, and to foster communication between the two sides to promote easier and more extensive analysis of data.

## Methods

### Operation

DEVEA was built as a Shiny application ^4^ in R ^3^ (V.4.1.1). Shiny is a package that facilitates the development of web application from R. It is particularly indicated for building interactive and user-friendly software wrapper.

The tool is hosted on a remote web server ( http://shiny.imib.es/devea), freely accessible. Apart from DEVEA’s public web server, the application can be used on a local computer (see the supplementary material for a detailed procedure as extended data here https://github.com/MiriamRiquelmeP/DEVEA/blob/main/Supplemental-Information.txt). Its source code is available on GitHub ( https://github.com/MiriamRiquelmeP/DEVEA), under the terms of the Apache license 2.0. DEVEA has been tested in Linux and Windows 10 operating systems locally, and has also been launched remotely with different browsers (Google Chrome, Mozilla Firefox and Internet Explorer). Locally running the application shares all of the same characteristics as the Shiny web application.

DEVEA relies on several existing R packages to carry out all the functionalities proposed (see supplementary material for the complete list). For instance, in order to handle the calculation of differentially expressed genes, the analysis is largely based on DESeq2 package. ^8^ The annotation is managed by the AnnotationDbi package, ^9^ collecting all the dedicated annotation databases for the different species (currently *Homo sapiens, Mus musculus* and *Rattus norvegicus*) for robust name conversion. For the enrichment calculation and visuals, the R packages topGO, ^10^ fgsea (Fast Gene Set Enrichment Analysis) ^11^ and clusterProfiler ^12^ together with other basic dependencies, such as ggplot2 ^13^ and plotly ^14^ has been used.

**Figure 1.**
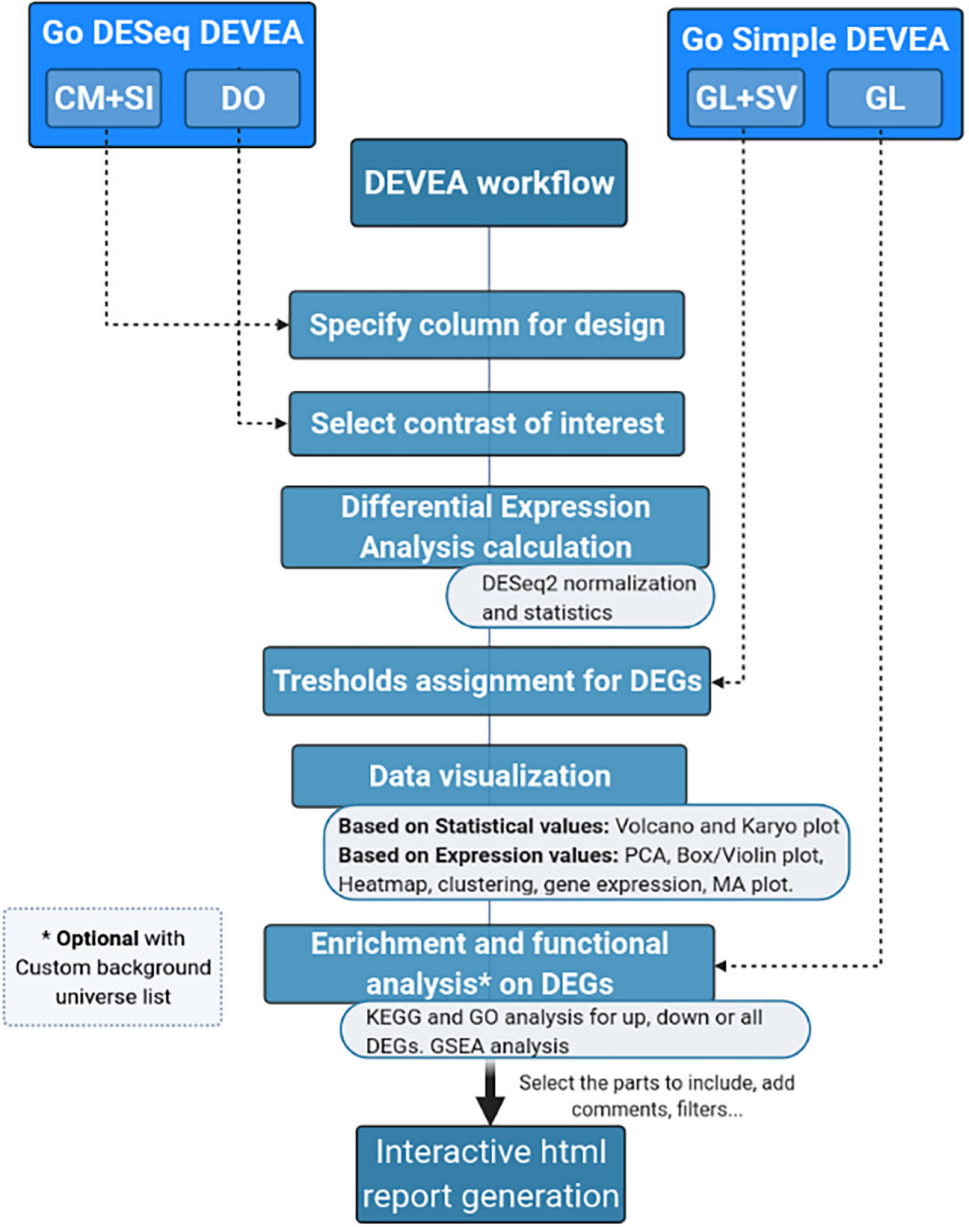
DEVEA global workflow.

The full DEVEA global workflow is shown on Figure 1. The main analysis path can be launched at different steps depending on the 4 input modes (check *Data requirement section* for details), represented at the top of the figure. The dashed arrows indicate where every input starts being incorporated in the flow, until the end of the workflow. More complex objects will generate more results. For each step of the analysis, intermediate results are available as tables and graphical representations in their dedicated spaces, detailed bellow in the circular notes. The vast majority of tables and plots are interactive, allowing the user to visualize data in real-time as well as to interact efficiently and can be individually downloaded. In the end, a global report can be generated and annotated. Each of these steps of the complete analysis will be described in their corresponding sections in the next paragraphs.

### Data requirement

**Figure 2.**
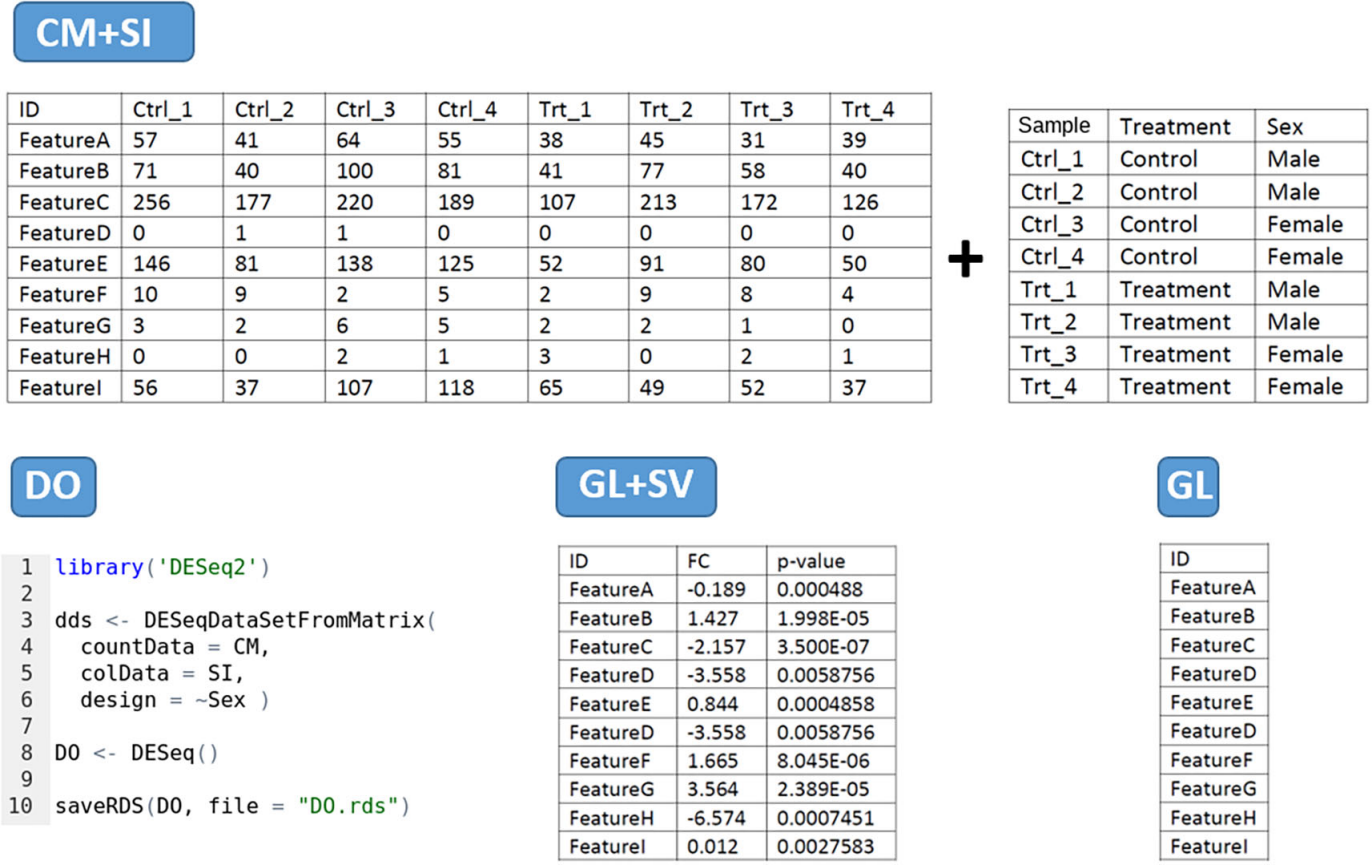
Possible data input formats for DEVEA. ’Feature’ represents a Gene Symbol or ENSEMBL gene ID.

The tool has four main data input modes ( Figure 2):

1. **CM + SI mode:** refers to a **counting matrix (CM)**, containing the raw number of counts per gene as round digits, where columns correspond to samples and rows to features. Data from other sources containing decimals must be rounded before being included in the tool. The CM should be associated with another file gathering the **sample information (SI)**, as a data frame comprising metadata about each sample, being the first column the identifier used by default as a label in the visualizations. This can be modified afterwards during the analysis. It should include any other relevant experimental factor (e.g. treatment/control, sex, cell type, tissue, etc). The design of the comparison will be determined by one of these factors. The column names in the CM and the first row names in SI must be equal, and the gene IDs in this file can be included in Symbol or ENSEMBL format. Both files can be in .CSV, .TXT or .XLSX format.
2. **DO mode:** based on a **DeseqDataSet object (DO)** generated by the *DESeq()* function from DESeq2 package. It is an object used to store the input values, intermediate calculations and results from a differential expression analysis. The user must have created it with the *CountData* field as the data matrix of counts, the *ColData* field with the sample information and a design formula specifying the experimental level to test for DEA. The first column in the CountData and the first row in the ColData are equal. The gene names can be included as gene Symbols or ENSEMBL gene IDs. The object must be compressed and extracted from R in .RDS format. If the differential expression object has been generated with a different tool or package, you may use the DEFormats ^15^ R function for a possible conversion.
3. **GL + SV mode:** a **gene list (GL)** with associated **statistical values (SV)** per gene. The first column should contain gene names (in Symbol or ENSEMBL format), the second column the fold-change and the third column the statistical adjusted p-value, in this precise order. Column names should be provided without special characters (i.e. GeneName, FC, padj). The values will be used without further modifications by DEVEA to set the threshold of expression change and the significance. This table can be uploaded in .CSV, .TXT or .XLSX file format.
4. **GL mode:** a **Gene list (GL)** consisting on a single column file in .CSV, .TXT or .XLSX format containing the gene names (in Symbol or ENSEMBL) and including a column name without special characters (i.e. GeneName, Genes, ID, etc). The gene list can be copied and pasted directly into then the dedicated field in the application, without the column name.

While the main data type for DEVEA’s usage is RNA-seq data, it is worth noticing that other types of “omics” can be used as input for the different modes. Obviously, the simple gene list (GL) can be build from any type of experiment, as long as the identifiers are recognized by the system. The more elaborated input data types, such as the counting matrix (CM + SI), can be built in some cases from different data where they can safely be processed by DESeq2 package. An example may be mass spectrometry data, that has become the method of choice for quantitative proteomics and can now assess protein samples with a respectable throughput. With label-free proteomics, it is possible to quantify proteins by using their spectral counts as an approximation of protein abundance, and then use statistical models such as DESeq2 although they are designed specifically for count data. In a study comparing different statistical methods for differential expression detection in label-free mass spectrometry proteomics, it was shown that DESeq2 performed well both in terms of detection of true positives as well as controlling for the number of false negatives. ^16^ Therefore it is perfectly possible to use this type of data in DEVEA, as long as the values represent unique measurements as integer numbers, and replacing the protein IDs by their coding gene name.

Another example could be the use of GL + SV mode with treated data from microarray analysis, where values such as FC of expression between groups and adjusted p-value are available from the signal intensity of the probes. It is highly recommended to work with log2FC and adjusted p-values.

### Implementation

***Getting started***

**Figure 3.**
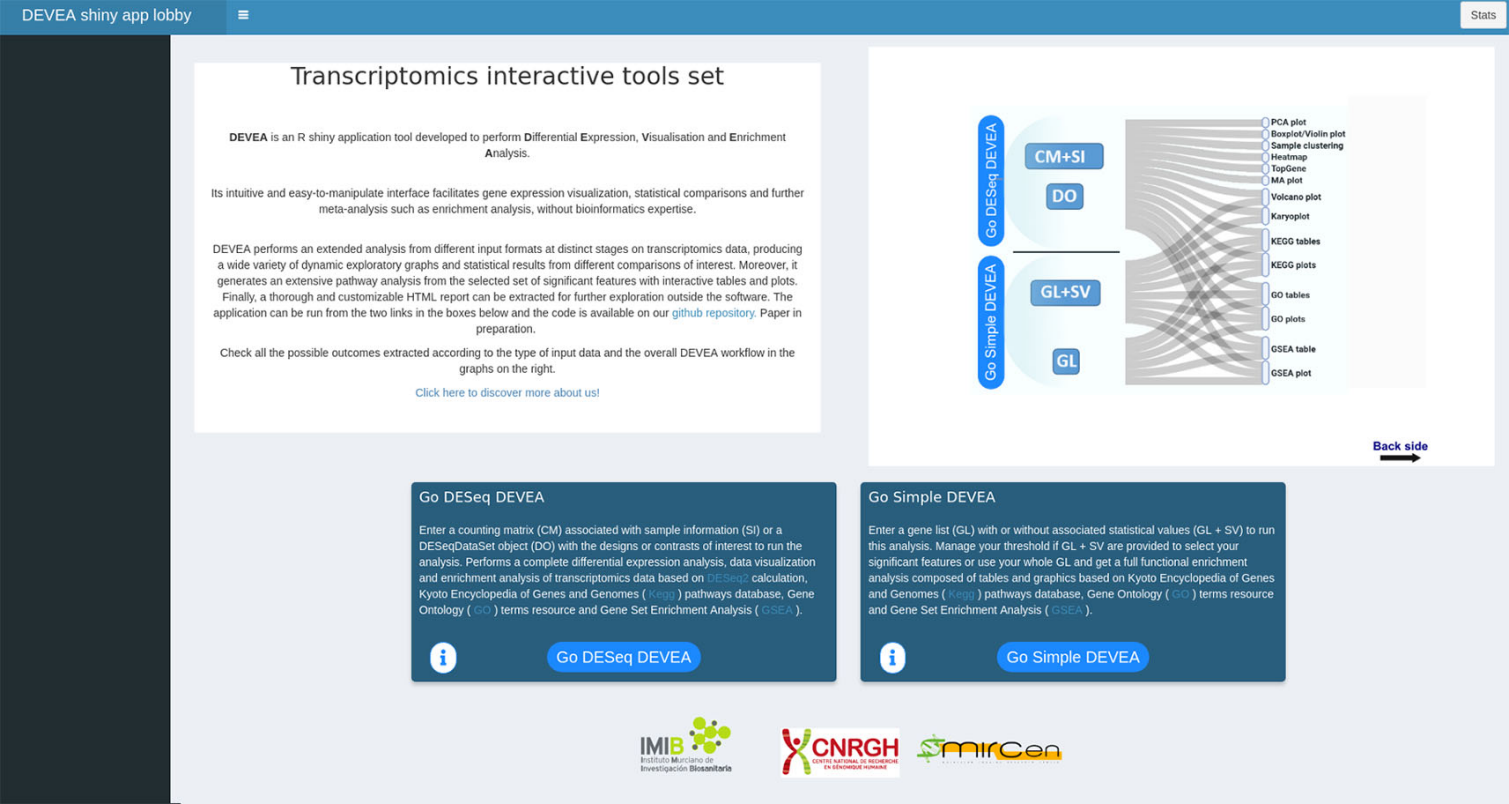
Generic screenshot of the lobby app showing the two possible pathways to start the analysis.

To start working with DEVEA, the adequate module to perform the analysis has to be chosen from DEVEA’s main lobby interface. The decision depends on the input data format. The user has to choose ‘Go DESeq DEVEA’ if their input is a CM + SI or a DO, whereas the ‘Go Simple DEVEA’ mode must be selected in the case of a GL + SV or a simple GL input files. See Figure 3 for a visual screenshot of the lobby.

***Data upload and statistical design specification***

**Figure 4.**
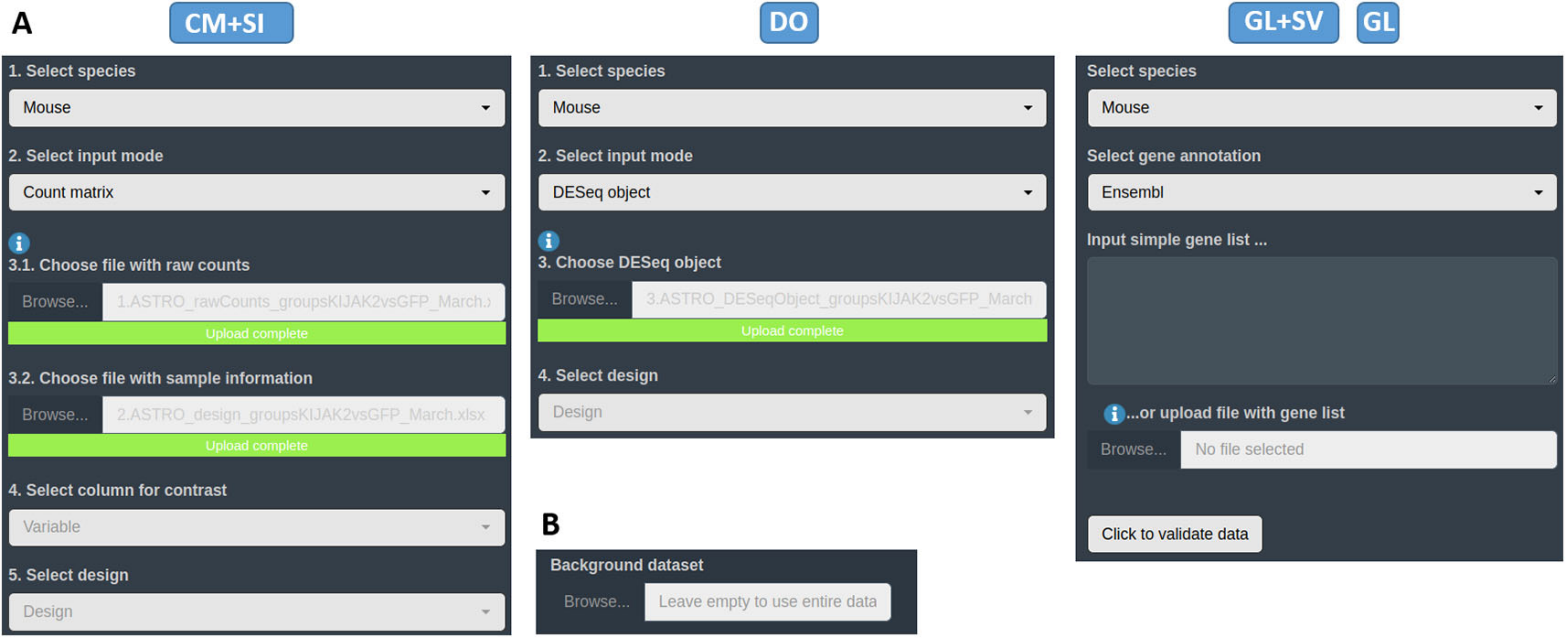
DEVEA’s import section. A: upload spaces for different types of input data. B: section to specify a custom background universe for EA.

Within the appropriate interface according to the data type, the first tab available corresponds to the ‘Input data’ section. The user has to upload their own data in one of the different accepted formats and types (see them on *Data requirement section*) in their dedicated spaces (see Figure 4A). For all input data, a field to specify the custom dataset to use as a background universe is available as well (see Figure 4B). If necessary for the user, some *demo data* representing the 4 different input types, can be found on GitHub https://github.com/MiriamRiquelmeP/DEVEA/Data. The nature of the *demo data* and how it was generated is described in the *Use case section.*

When a CM + SI or a DO are used as input data, it is important to indicate the statistical design or contrast for the expected comparison. The design formula expresses the variables which will be used in modeling to calculate the differential expression in following steps. For the CM + SI input format, the levels of interest that will compose the final design must be included in one of the columns of SI file. By entering the column name in the ‘Select column for contrast’ field, the program extract the conditions and calculates the combination based on the distinct levels (i.e. *Treatment_Control_vs_Treatment* if column Treatment is selected or *Sex_Male_vs_Female* will be displayed if Sex is selected from SI in Figure 2 - CM + SI). In cases where more than 2 levels are available, the application will propose all 1 vs 1 combinations of them. Then, the most relevant combination for the analysis has to be selected by the user in the ‘Select design’ part.

With a DO data type, the column for the design must have been already incorporated when generating the DESeq2 object in R. Only simple designs will be generated and/or can be selected from ‘Select design’ field (i.e. *Sex_Male_vs_Female* if *design = Sex* is specified in the formula as in Figure 2 - DO).

***Differential expression analysis (DEA) and data view***

The first key performance of the application consists in extracting the descriptive information based on the feature expression and the statistical contrast representing the differential expression analysis part. In CM + SI data type a new DeseqDataSet object is calculated from the files and information provided by the user. In DO or GL + SI input modes, the application retrieves the important values already included in the objects. All transformations, normalization and measurements applied to the data at this step are performed with functions included in DESeq2 R package. It should be noted that DEA calculation is not possible with the simple GL input mode, due to the lack of expression values and statistical details. Following comments will not apply to this object.

**Figure 5.**
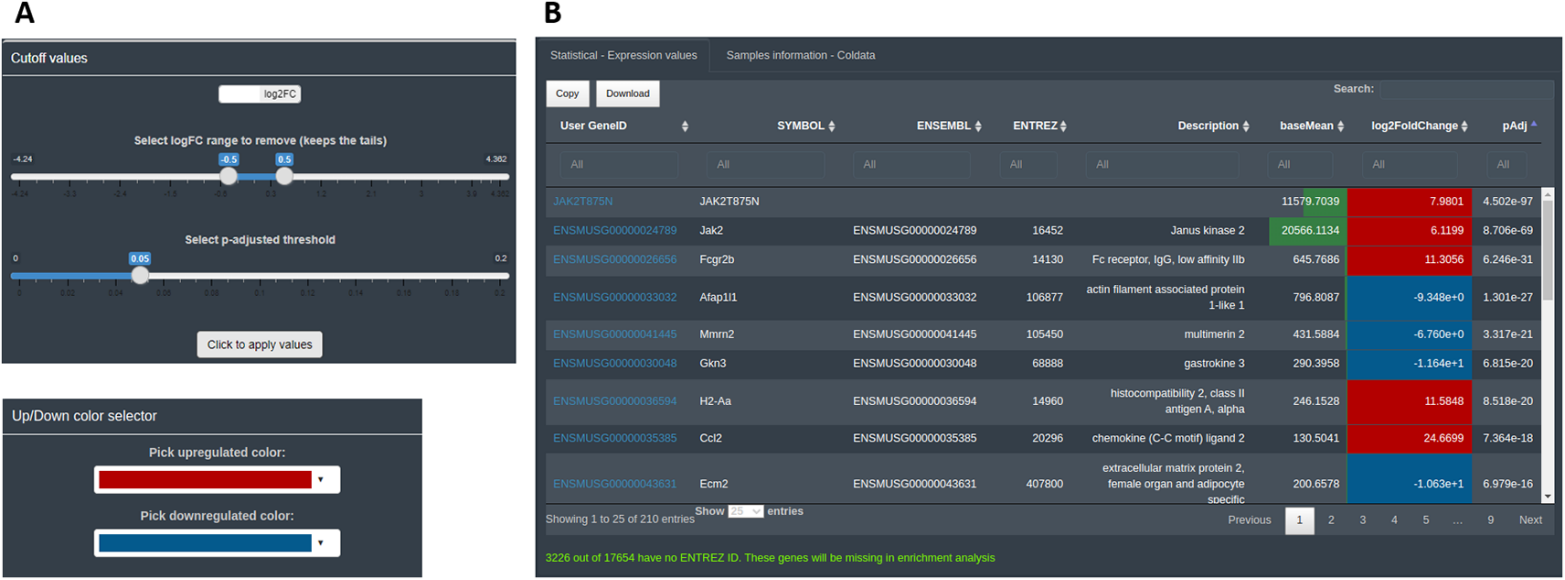
Basic data visualization for DEGs in DEVEA. A: controls used to set the statistical thresholds and cosmetics parameters. B: interactive result table.

The calculations and the statistical results are accessible in the ‘Preview dataset’ section tab. The user can explore at this step of the analysis the number of features considered as differentially expressed and their direction, and establish the descriptive statistics thresholds to consider them DEGs. By default, DEVEA uses prefixed log fold change|lfc|> 0.5 and p-value < 0.05 thresholds, that can be adapted by the user at any moment and thus modulate the list of DEGs. Moreover, the information uploaded and the descriptive statistics will be used to establish and control some interactive parts of the plots. For example, the color of defined up-regulated or down-regulated genes can be chosen ( Figure 5A). For the first two input objects (CM + SI and DO), a complete table of results, named ‘Statistical - Expression values’, is displayed showing useful information such as base means across samples, log2 fold changes, standard errors, raw and adjusted p-values for the specific design selected. A second table is also shown with the DEA analysis details called ‘Samples information - Coldata’ ( Figure 5B). For the GL + SV mode, raw data are displayed in a table. The user can monitor gene name conversion, explore values interactively and sort, filter and download them at any time.

**Figure 6.**
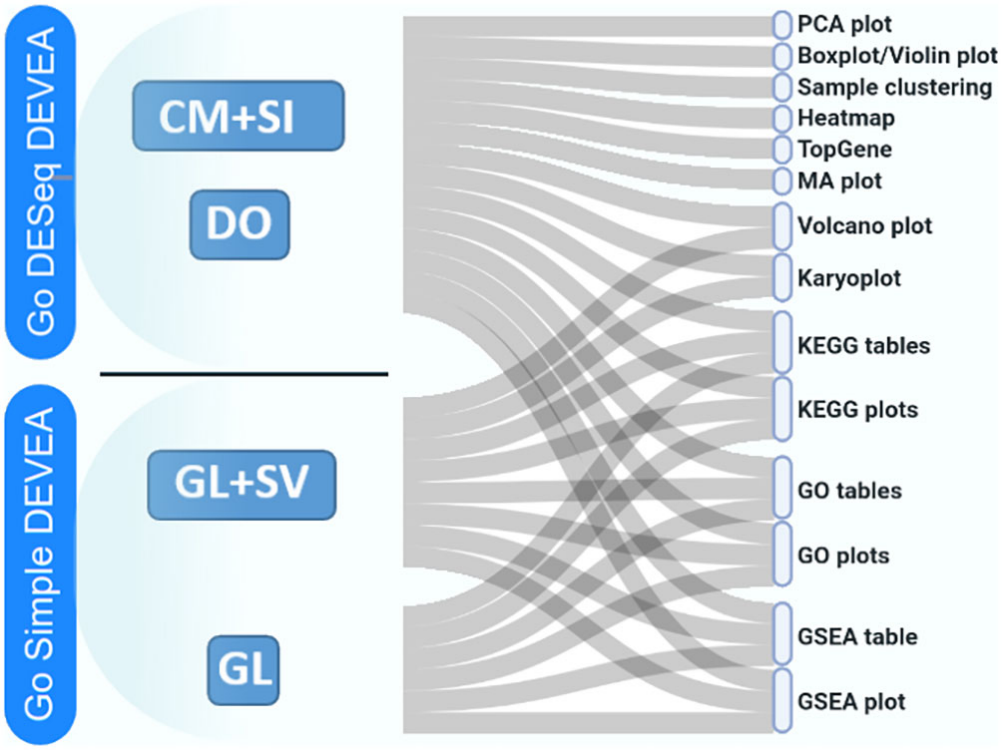
Possible types of graphical representations in DEVEA depending on data input type.

**Figure 7.**
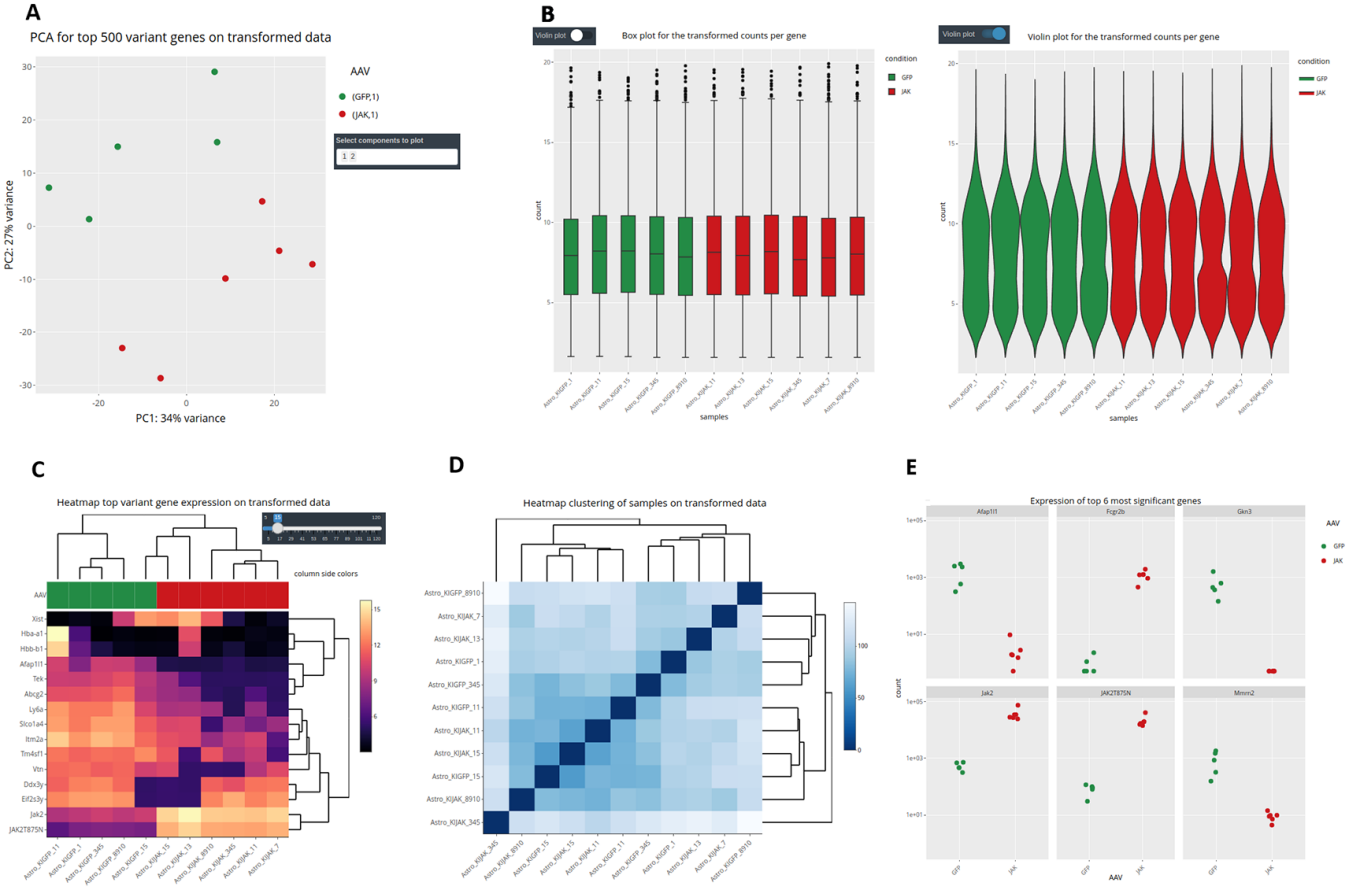
Advanced data visualization in DEVEA. A: PCA plot. B: box and violin plots. C: gene expression heatmap. D: sample hierarchical clustering. E: dot plots.

**Figure 8.**
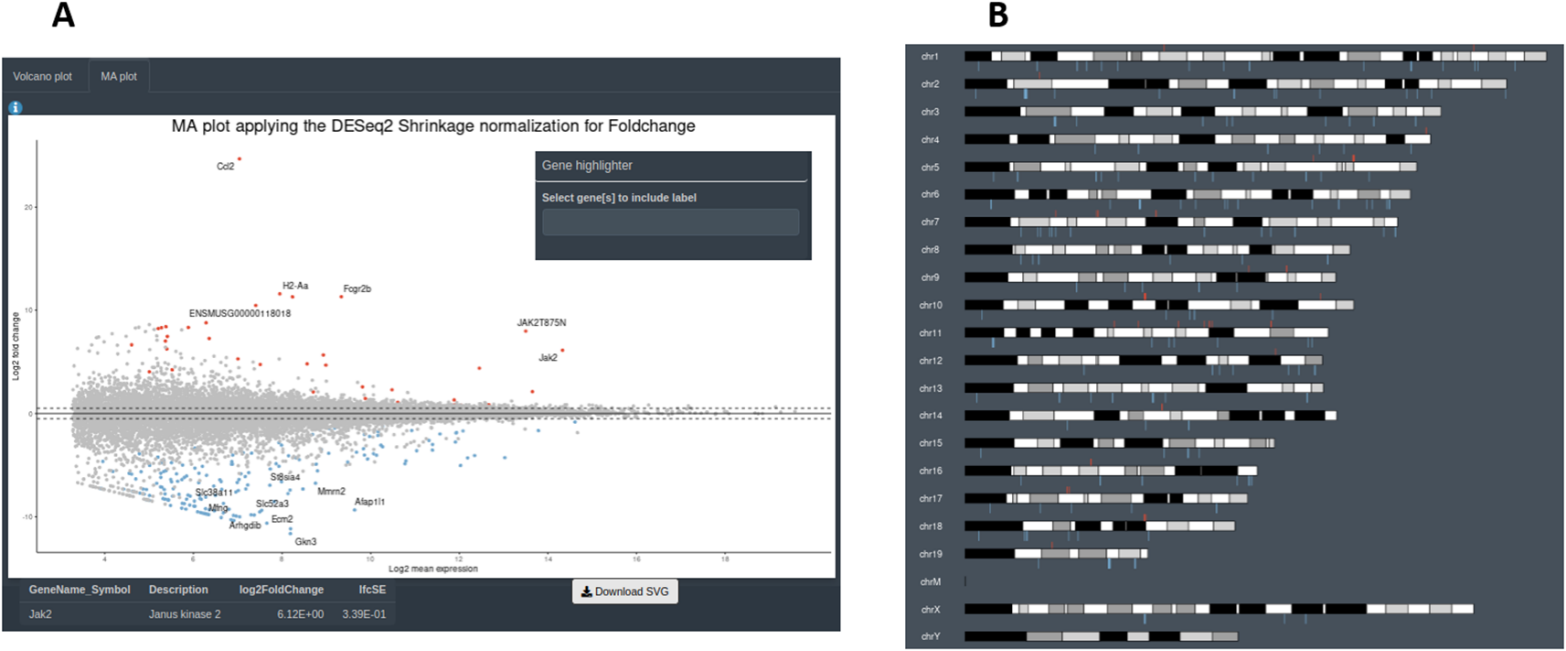
Additional data visualisations available from DEVEA. A: section to explore volcano and MA plots. B: karyotype plot.

It is important to stress that, as different elements can be extracted from the distinct input objects depending on their complexity, the number of graphs available for each of them will vary ( Figures 1 and 6). Using the raw expression values that are available only in CM + SI and DO input modes, the user can explore data in a Principal Component Analysis plot (PCA) with the top 500 variant features, to show clusters of samples based on their similarity selecting the principal components of interest; a box or violin plot for gene expression distribution across the dataset; a heatmap representation of the top variant genes, regulated by the user; and a dot plot with the expression of the top 6 variant genes or a selection of individual genes of interest ( Figure 7). A second group of graphs represents the plots that allow to visualize genes related only to their statistical values. This can be displayed from CM + SI, DO and GL + SV modes. They consist in a volcano plot, that shows statistical significance (adjusted p-value) versus the magnitude of change (FC) regarding the contrast levels; and a karyotype plot showing the DEG position on the genome and the direction of change (color coded by up- or down-regulated) ( Figure 8B). Finally, it is also possible to combine gene expression with statistical values, from CM + SI and DO input modes, to generate a MA plot (an application of a Bland–Altman plot for visual representation of genomics data ^17^) ( Figure 8A) displaying feature labels and statistical values by clicking on each gene dot.

***Enrichment analysis (EA) and visualization***

The last stage of the analysis with DEVEA is EA. This is a method to identify classes of defined categories that are over-represented in the list of DEGs. These categories may be associated with disease phenotypes, biological pathways, or cellular functions. DEVEA uses the differentially expressed and significant features to retrieve the over-represented terms from several well known databases. This major block of the DEVEA analysis can be carried out from all data input types. It consists of an extensive EA after the selection of appropriate statistical values for defining DEGs from CM + SI, DO and GL + SV, or using all components included in the simple GL input. It collects significant terms from KEGG (Kyoto Encyclopedia of Genes and Genomes ^18^) and GO (Gene Ontology ^19^) Biological process, Molecular function and Cellular component databases. Furthermore, a GSEA-type analysis (Gene Set Enrichment Analysis ^20^) can be performed from different databases for the whole set of features. In the case of CM + SI, DO and GL + SV input modes, KEGG and GO analyses are performed for all DEGs together, and for the subset of up- and down-regulated genes in separated tabs. GSEA analysis is always performed on the complete set of genes and uses the statistical values associated with the features. With the simple GL data input, enrichment can only be performed for the whole set of genes for KEGG and GO analysis and no GSEA analysis will be possible, due to the lack of statistical information.

**Figure 9.**
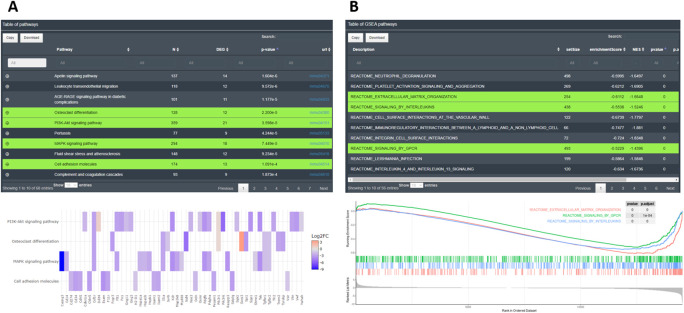
Examples of enrichment analysis with DEVEA. A: KEGG EA table and heatmap. B: GSEA-type EA table and plot.

In the EA performed by DEVEA, the main results are shown as interactive tables containing detailed information on the enrichment from each database. In KEGG and GO categories, the tables display columns for the name of the significant pathways or terms, their (adjusted) p-values and additional descriptive information such as total number of genes associated or the DEGs participating in the pathway. The user can also display the feature names that match in the pathway from the + symbol. Below each table, additional plots can be created by selecting rows of interest (showed in green on the Figure 9A). The plots are interactive and reactive. They can be changed at any time by selecting new lines in the table. The user can visualize results as word cloud, circle plot, bars plot, chord plot, dot plot, heatmap or net plot representing different elements from the tables. For GSEA-type analysis, the results are displayed as a table containing the significant enriched pathways from the selected databases. In the table, important GSEA calculation parameters are available and below a typical GSEA plot is shown from the lines selected in the table (see Figure 9B).

As a special feature offered by DEVEA, a custom gene list can be loaded to be used as the background gene universe for EA ( Figure 4B). For example, the user can use a background list containing only expressed genes in this experiment. It will control for experimental context and may enable the results to define not only the nature of the samples but also representative functions. This is especially important to consider in microarray studies when a limited number of probes is used, or in studies of specific cells or tissue with a restricted set of detectable genes.

For this EA, internal ENTREZ ID gene code is used to associate gene names in Symbol or ENSEMBL with the enrichment annotation in KEGG, GO and GSEA libraries. An exhaustive conversion across different functions is conducted to retrieve all possible terms, since they are not registered in all conversion databases similarly. Furthermore, not all genes are curated or annotated and therefore some will be left out of the EA (the portion of genes is indicated in the application below the ‘preview table’ as shown in Figure 5B). This is a limitation from the automated databases conversions without manual curation. For this reason, the number of species is limited and not all genes will be used for the enrichment part, but the results obtained are more robust.

***Global report***

**Figure 10.**
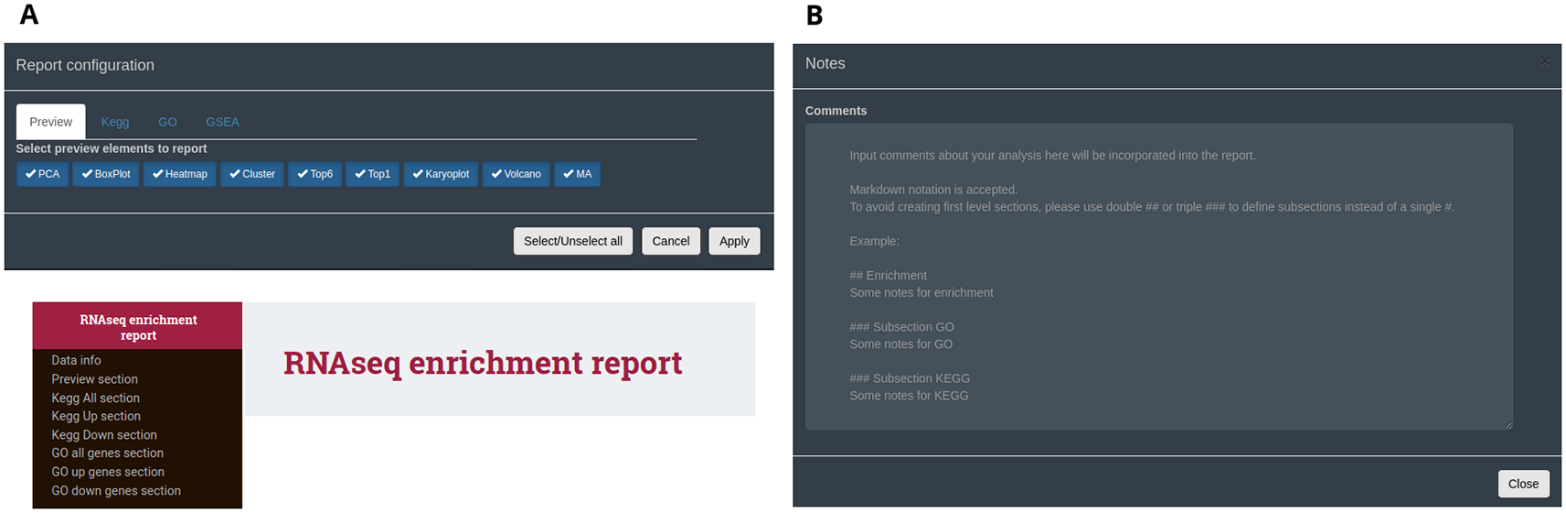
Interactive final DEVEA report functionalities and features. A: configuring box and final HTML report. B: notes transferable to the report.

An interactive HTML report can be generated from all data types following analysis and exploration with DEVEA. It is available in the ‘HTML report’ button at the top fixed part of the application. To create it, the user can select the individual set of figures, tables and results to be kept in this single html document ( Figure 10A). Plots will retain the last aesthetic indicated in the graphical parameters (e.g. colours, shapes, labels, terms). Only full tables will be kept, without taking into account potential filters applied during the analysis, allowing full exploration, sorting and re-filtering of the whole data outside of the application. It should be noted that the majority of the results can also be copied or downloaded in high-quality format at any step of the analysis inside DEVEA. Importantly, comments can be included at any step of the analysis in a dedicated section at the top right position of the application, and will be automatically saved ( Figure 10B). They can be displayed in the final report to ensure that the observations made throughout the analysis, with special interpretations or results are maintained.

Please note that if any of the graphs or tables do not work in the application, when there are not enough results, genes or routes to display them, the report cannot be generated with errors. Unselect the problematic plots or tables to be able to render the rest of the report.

## Use case

**Figure 11.**
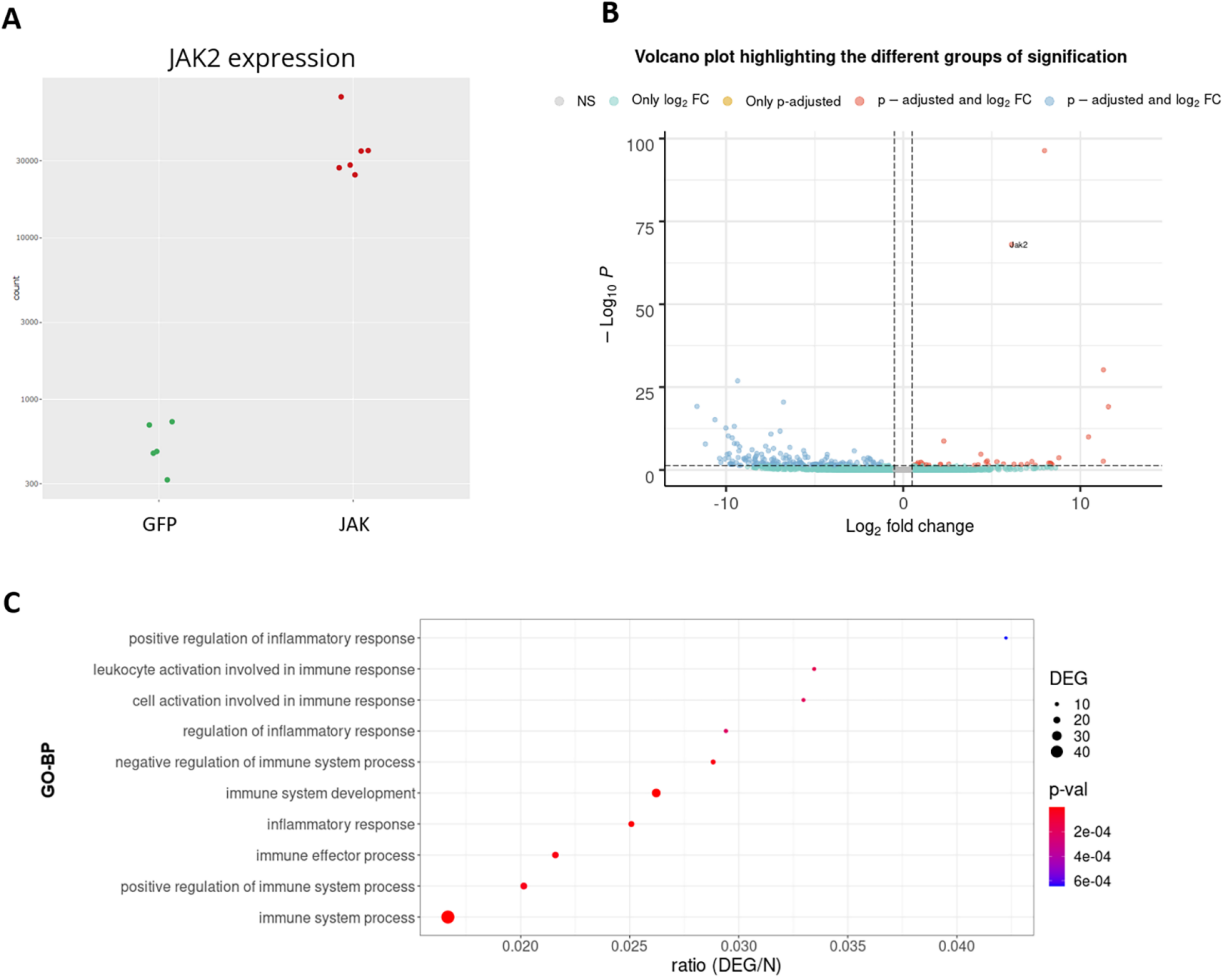
RNA-seq analysis of the JAK2/STAT3 pathway in astrocytes of a Huntington’s disease model mouse. A: expression levels of the *Jak2* gene between groups. B: volcano plot. C: GO-BP enriched categories.

To demonstrate the usefulness of DEVEA, a RNA-seq dataset generated by our team and published recently ^21^ was investigated using the application. The study aimed to characterize the role of JAK2/STAT3 signaling in astrocytes in the context of Huntington’s disease (HD). HD is a rare genetic neurodegenerative disease leading to severe motor, cognitive and psychiatric symptoms, with no curative treatment available. ^22^ Astrocytes, an heterogeneous group of star-shaped glial cells perform key functions in the brain, they provide nutrients to neurons, regulate synaptic transmission, and contribute to nervous tissue repair following injury. ^23^ Astrocytes become reactive in the brain of HD patients and their impact on HD progression is still unclear. ^24^ The study used a genetic mouse model of HD. Treated mice were injected with an adeno-associated viral (AAV) vector targeting astrocytes and encoding a constitutive form of the JAK2 kinase (JAK2T875N) to activate the JAK2-STAT3 pathway. Control mice were injected with a similar AAV expressing GFP. Astrocytes were isolated and sequenced by RNA-seq from Illumina platform. Reads were aligned on the mouse genome (ENSEMBL GRCm38 release 96) and a count matrix was generated. Data were adapted and integrated as different input objects in DEVEA to test all functionalities. For instance, Figure 7A shows that control (GFP, N=5, in green) and treated (JAK, N=6, in red) samples are clearly separated on a PCA plot, with a better separation achieved on PC2 (representing 27% of the total variance). Figure 7C- D also show two types of clustering profile, which group samples from the same group together and display genes with higher variability. Figure 11A shows that the levels of *Jak2* are higher in treated (JAK) versus control (GFP) groups, as expected by AAV-mediated gene transfer (log2(FC) = 6, associated adjusted p-value 8.7E-69). In addition, a volcano plot demonstrates that the JAK2T875N causes down-regulation of many genes DEGs, shown in blue in the upper left quartile of the graph on Figure 11B. Finally, EA in the treated (JAK) versus control (GFP) list of DEGs shows that many GO-BP terms are related to Immunity/Inflammation, a process linked to the reactive changes in astrocytes induced by JAK2 ( Figure 11C).

All data corresponding to this research project are available under the four different DEVEA input types. They are available on the GitHub web site (see the *Software Availability section*). They consist in (1) a set of CM + SI, where features are genes in the ENSEMBL format and expression represents raw counts after alignment and filtering to remove non-expressed genes from the CM. The final number of features is 18260 + 2 custom genes representing the two transgenes JAK2T875N and GFP). The SI file contains all relevant information on sample characteristics; (2) a complete DO built from the same CM and SI files with a design based on the comparison of the two different AAVs (design = AAV); (3) a GL + SV file manually created according to the *DESeq2* results. The file is generated with 3 columns that contains the gene names, FC and P-Value for the top 500 most significant genes and (4) a unique GL containing only the 268 DEGs from the comparison of interest, reported from the DESeq2 analysis with adjusted P-Value < 0.1 statistical threshold and no threshold for the FC (for further details on the input data, see the *Data requirement section*).

With this user case, no errors were detected throughout the analysis. DEVEA, thanks to a large range of graphics and statistical analysis highlighted significant differences between reactive astrocytes in the JAK group and control astrocytes in the GFP group. Thanks to extensive EA analysis, DEGs were associated with important biological functions such as immunity/inflammation pathways as well as cytokine signaling, proteostasis and energy metabolism. These results are consistent with the insight described earlier this year in the team. ^21^

## Related works (state-of-the-art)

The field of application tools for transcriptomics data visualization, DEA and EA is constantly growing. To compare DEVEA functionalities with similar applications, six recently published tools that operate through a graphical user interface were selected, provide interactive results, and are based on stable and maintained R packages. The six selected tools are iDEP, ^5^ ShinyGO, ^25^ DEGenR, ^26^ GENAVi, ^6^ RNfuzzyApp ^27^ and ideal. ^7^ A detailed comparison of the DEVEA main attributes is shown in Table 1. Characteristics that are related to data management and import into the application has been stressed at the beginning, followed by the various modes of DEG identification and different interactive graphical results, EA calculations and global reporting and hosting. It is clear from Table 1 that most of the selected application share many functionalities with DEVEA. One exception is the application ShinyGO, which offers significantly less options since it is designed to perform uniquely enrichment calculations from simple gene lists. However, some other specific differences exist with the rest of the tools closer in purpose to the one presented here. DEVEA has more flexibility in terms of data type import into different formats, representing different stages of the analysis. Similarly, the user has a wide range of exploration possibilities via GL and GL + SV input formats, in terms of data generation and origin. The list of features and the associated statistics may have been generated from many different external tools or could represent several analysis types as long as they are eventually converted into gene names (i.e. Microarray data results, proteomics results, gene lists from the literature, etc.). As a further advantage, the ability to import complex objects, such as DO increases the number of visualization and analysis options. Despite this, not all possible visuals that can be generated from these objects are included in the application, which thus has room for expansion. In particular, DEVEA could further develop data management functionalities, by extending the capacity of dealing with batch effect or data pre-processing and filtering. One of the possible drawbacks is the low number of available species for the EA compared with other tools or the potential mismatches when converting gene names. To counter this, numerous graphics are displayed with whom the user can interact and perform a complete EA. The EA can be generated from all DEGs, either split by the direction of expression change, or merged into a single list. The custom global report is a unique feature to DEVEA. This might be very handy to share results with collaborators, because the user can easily insert comments, and transfer the fully-interactive HTML report. Last, DEVEA appears slightly more flexible in terms of the application hosting and running, since it is possible to run it online (DEVEA web server), or offline (from R). For instance, some applications like DEGenR and RNfuzzyApp do not offer the possibility to run the application online.

**Table 1.** DEVEA functionalities compared to similar software tools in their online version.

Publication year	DEVEA	iDEP ^5^	ideal ^7^	GENAVi ^6^	RNfuzzyApp ^27^	DEGenR ^26^	ShinyGO ^25^
	2022	2018	2020	2019	2021	2021	2020
**IMPORT DATA/MANAGEMENT:**							
**Count data input mode**	x	x	x	x	x	x	
**DESeq object input mode**	x						
**Gene list input mode**	x						x
**Several gene names**	x	x	x	x	x	x	x
**>2 species**	x	x	x		x		x
**Raw data accessibility**	x	x	x	x	x		
**Demo data**	x	x	x	x			x
**DEA COMPUTATION & DATA VISUALIZATION:**							
**DEA statistical calculation**	x	x	x	x	x	x	
**Manage stats threshold**	x	x	x	x		x	
**Interactive statistics summary table**	x		x	x	x	x	
**Interactive preview visuals**	x	x	x	x	x	x	
**Interactive DE visuals**	x	x	x	x	x	x	
**ENRICHMENT ANALYSIS & VISUALIZATION:**							
**Custom background universe**	x						x
**Split results by DE directions**	x		x		x		
**KEGG results**	x	x		x	x	x	x
**GO results**	x	x	x	x	x	x	x
**GSEA type results**	x	x	x			x	
**Interactive EA tables**	x	x	x	x	x	x	x
**Interactive EA visuals**	x	x	x	x	x	x	x
**DOWNLOAD & REPORT:**							
**Individual plots download (.SVG, .HTML, .PNG)**	x	x	x	x	x	x	x
**Interactive report**	x		x	x			
**Custom report**	x						
**ACCESSIBILITY:**							
**Public server**	x	x	x	x			x
**Source code available**	x	x	x	x	x	x	x

Although overall applications share common analysis blocks, DEVEA presents more graphical variety than most of them. For example, as a criteria to consider in the table that they contain “Interactive preview visuals” to preliminary explore the data, only one of PCA plot, violin/box plot of sample profile, heatmap for gene expression, sample clustering, gene expression dots plot per groups should be generated from the applications. For “Interactive DE visuals” of the DEA statistics and profile the criteria consist of including at least one of volcano plot, MA plot or karyoplot. Finally, for the “Interactive visuals” to navigate the EA, the marked tools include at least one of bars plot, dots plot, chord plot, heatmap, net plot, word cloud, circle plot of the enriched terms. For most applications, only a small subset of these plots are implemented. DEVEA contains all these graphs, requirement that none of the others met.

## Conclusion and future directions

The DEVEA application is developed to improve the set of existing software to perform DEA, data visualization and annotation or EA from transcriptomics data. DEVEA meets the need for applications that give sufficient usage autonomy, without compromising the complexity and accuracy of the results. It provides an interactive and user-friendly interface accessible for users without bioinformatics training, with high diversity of analysis components. Researchers can explore their data, carry statistical DEA and subsequent EA from distinct well-known databases without losing possible customization in real-time. It is a wide wrapper of functions in a single tool, avoiding the use of different tools/websites to run the distinct steps of the transcriptomics analysis and to reach this level of advanced ready-to-publish visuals, tables and results. One of the main strength is the incorporation of several input data type options. The possibility to include a custom background make DEVEA suitable for analysis in which correction of some experimental bias could lead to better results in the EA part. Another key advantage is that DEVEA let the user extract their results individually or in a still interactive way through a custom HTML format file. To further develop DEVEA analyses, we plan to later offer additional pre-treatment options (remove batch effect, filter genes by expression, etc.). More species can also be integrated. Other improvements include transcription factor enrichment analysis and use of complete datasets from different omics such as proteomics spectral counts matrix, microarray expression matrix, etc. In conclusion, a purpose of DEVEA is to promote a dialogue between biologists and bioinformaticians, particularly to produce suitable data and to understand the validity of the data needed to create the best downstream results.

## Software availability

1. Software available at http://shiny.imib.es/devea/. Archived source code as at time of publication: https://doi.org/10.5281/zenodo.6657245.
2. Latest source code on https://github.com/MiriamRiquelmeP/DEVEA.
3. Test files for every input mode can be found also on https://github.com/MiriamRiquelmeP/DEVEA Data section.
4. Tutorial accessible from both DEVEA modules (DESeq DEVEA and Simple DEVEA) in the ‘Tutorial’ section from the top controls and independently on https://shiny.imib.es/DESeqDevea/tutorial.html or https://shiny.imib.es/simpleDevea/tutorial.html.

License: Apache license 2.0.

## Author contributions

MRP and FPS conceived the application, did the development and wrote the manuscript. CE, SB and EB supervised the work, tested the application and wrote the manuscript. JFD contributed fund acquisition and resources for the project. All authors discussed the results and contributed to the final manuscript.
