# DEVEA: an interactive shiny application for Differential Expression analysis, data Visualization and Enrichment Analysis of transcriptomics data

**DOI:** 10.12688/f1000research.122949.2

**Published:** 2023-03-24

**Authors:** Miriam Riquelme-Perez, Fernando Perez-Sanz, Jean-François Deleuze, Carole Escartin, Eric Bonnet, Solène Brohard

**Affiliations:** 1 Université Paris-Saclay, CEA, CNRS, MIRCen, Laboratoire des Maladies Neurodégénératives, Fontenay-aux-Roses, 92265, France; 2 Centre National de Recherche en Génomique Humaine (CNRGH), Institut de Biologie François Jacob, CEA, Université Paris-Saclay, Evry, 91000, Evry, France; 3 Biomedical Informatics & Bioinformatics Service, Institute for Biomedical Research of Murcia (IMIB), Murcia, 30120, Spain

**Keywords:** Bioinformatics, transcriptomics, RNA sequencing, differential expression analysis, enrichment analysis, visualization, R, Shiny, interactive reports.

## Abstract

We are at a time of considerable growth in transcriptomics studies and subsequent *in silico* analysis. RNA sequencing (RNA-Seq) is the most widely used approach to analyse the transcriptome and is integrated in many studies.

The processing of transcriptomic data typically requires a noteworthy number of steps, statistical knowledge, and coding skills, which are not accessible to all scientists. Despite the development of a plethora of software applications over the past few years to address this concern, there is still room for improvement.

Here we present DEVEA, an R shiny application tool developed to perform differential expression analysis, data visualization and enrichment pathway analysis mainly from transcriptomics data, but also from simpler gene lists with or without statistical values.

The intuitive and easy-to-manipulate interface facilitates gene expression exploration through numerous interactive figures and tables, and statistical comparisons of expression profile levels between groups. Further meta-analysis such as enrichment analysis is also possible, without the need for prior bioinformatics expertise.

DEVEA performs a comprehensive analysis from multiple and flexible data sources representing distinct analytical steps. Consequently, it produces dynamic graphs and tables, to explore the expression levels and statistical results from differential expression analysis. Moreover, it generates a comprehensive pathway analysis to extend biological insights. Finally, a complete and customizable HTML report can be extracted to enable the scientists to explore results beyond the application. DEVEA is freely accessible at https://shiny.imib.es/devea/ and the source code is available on our GitHub repository https://github.com/MiriamRiquelmeP/DEVEA.

## Introduction

RNA sequencing (RNA-seq) has become a routine and popular technique for genome-wide and transcriptomics expression analysis. ^1^ As a result, techniques analyzing RNA are extensively incorporated in basic science research and are even increasingly used as molecular diagnostics for human health. These may include diagnosis, prognosis and therapeutic selection. ^2^

However, in order to leverage the full power of this technique, several stages and tools are necessary to translate expression profiles into valuable outcomes. The R statistical environment ^3^ provides many well-known packages to perform key steps of a complete RNA-seq analysis pipeline. Possible examples include differential expression analysis (DEA) functions, leading to lists of differentially expressed genes (DEGs), and annotation enrichment analysis (EA, sometimes called pathway analysis) libraries, which will identify biological pathways or cellular functions significantly enriched from the list of DEGs.

Nevertheless, most of these powerful packages are command-line based or demand coding knowledge and are therefore out of reach for scientists with limited computational training. Besides, analyses can be started at different points in the workflow, from raw or partially analyzed data from different tools, to individual lists of favorite final features. Thus, tools providing several ways to start an analysis are more flexible than others using a single data type. Providing flexible user-friendly tools for the analysis and visualization of gene expression data can help researchers to move from high-throughput genomics to basic scientific research. To bridge this gap, an increasing number of software tools are being released, based on intuitive, point-and-click, graphical interfaces. Frameworks such as R Shiny, ^4^ an R package, facilitate the creation and release of interactive web tools. Certain RNA-seq analysis applications from the literature may include iDEP, ^5^ GENAVi ^6^ and ideal, ^7^ among others.

However, there are still ways to improve the functionalities of these tools. For instance, supporting input data types of different levels of complexity can extend the level of performance of the tool. It is also essential to include widely used types of analyses such as differential expression and pathway analysis, with enough options for calculations and graphical representation to generate valuable results. At last, user-friendliness is a particularly important point since these tools aim at helping the non-specialist. Therefore providing a robust and easily accessible web interface is an essential asset. With these considerations in mind, we have developed DEVEA, a new interactive R Shiny application for DEA, data exploration, data visualization and functional EA. DEVEA provides an easy-to-use interface to load data in various formats and complexities according to the stage of the analysis, including raw RNA-seq count data, pre-analyzed data, simple lists of genes or proteins obtained from different sources, with or without statistical values associated. From these different types of input data, it generates a wide set of dynamic plots and tables allowing quick navigation through gene expression profile or enrichment analysis results. The outputs can be downloaded easily and the user can create custom and operable reports in HTML format. DEVEA is implemented as a publicly available web server and can be optionally downloaded to be used locally. DEVEA aims to conduct a proper analysis by reaching out to both life scientists (gathering the biological expertise) and bioinformaticians (offering the technical expertise), and to foster communication between the two sides to promote easier and more extensive analysis of data.

## Methods

### Operation

DEVEA was built as a Shiny application ^4^ in R ^3^ (V.4.1.1). Shiny is a package that facilitates the development of web applications from R. It is particularly indicated for building interactive and user-friendly software wrappers.

The tool is hosted on a remote, freely accessible web server ( http://shiny.imib.es/devea). Apart from DEVEA’s public web server, the application can be used on a local computer (see the supplementary material for a detailed procedure here https://github.com/MiriamRiquelmeP/DEVEA/blob/main/Supplemental-Information.md). Its source code is available on GitHub ( https://github.com/MiriamRiquelmeP/DEVEA), under the terms of the Apache license 2.0. DEVEA has been tested in Linux and Windows 10 operating systems locally, and has also been launched remotely with different browsers (Google Chrome, Mozilla Firefox and Internet Explorer). However, for the best user experience in terms of rendering and visualization, we recommend to use Mozilla Firefox. Other browsers may present display issues when deploying some of the elements of the application and generate errors. Locally running the application shares all the same characteristics as the Shiny web application. A comprehensive guide on how to use the application from the different input modes can also be explored through the accessible tutorial from both DEVEA modules (DESeq DEVEA and Simple DEVEA) in the ‘Tutorial’ section from the top controls and independently on https://shiny.imib.es/DESeqDevea/tutorial.html or https://shiny.imib.es/simpleDevea/tutorial.html.

DEVEA relies on several existing R packages to carry out all the functionalities proposed (see supplementary material for the complete list). For instance, in order to handle the calculation of DEGs, the analysis is largely based on DESeq2 package. ^8^ The annotation is managed by the AnnotationDbi package, ^9^ collecting all dedicated annotation databases for the different species (currently *Homo sapiens, Mus musculus, Rattus norvegicus* and *Arabidopsis thaliana*) for robust name conversion. For the enrichment calculations and visuals, the R packages topGO, ^10^ fgsea (Fast Gene Set Enrichment Analysis) ^11^ and clusterProfiler ^12^ together with other basic dependencies, such as ggplot2 ^13^ and plotly ^14^ have been used. All those packages are widely used by the community and well maintained. We have selected DESeq2 for differential expression analysis as it is one of the most popular and well-tested tools for this task. Furthermore, it was shown ^15^ that DESeq2 has a good overall performance regardless of presence of outliers and proportion of DE genes compared with other methods, such as Limma-Voom, ^16^ BaySeq ^17^ and edgeR ^18^ among others.

**Figure 1.**
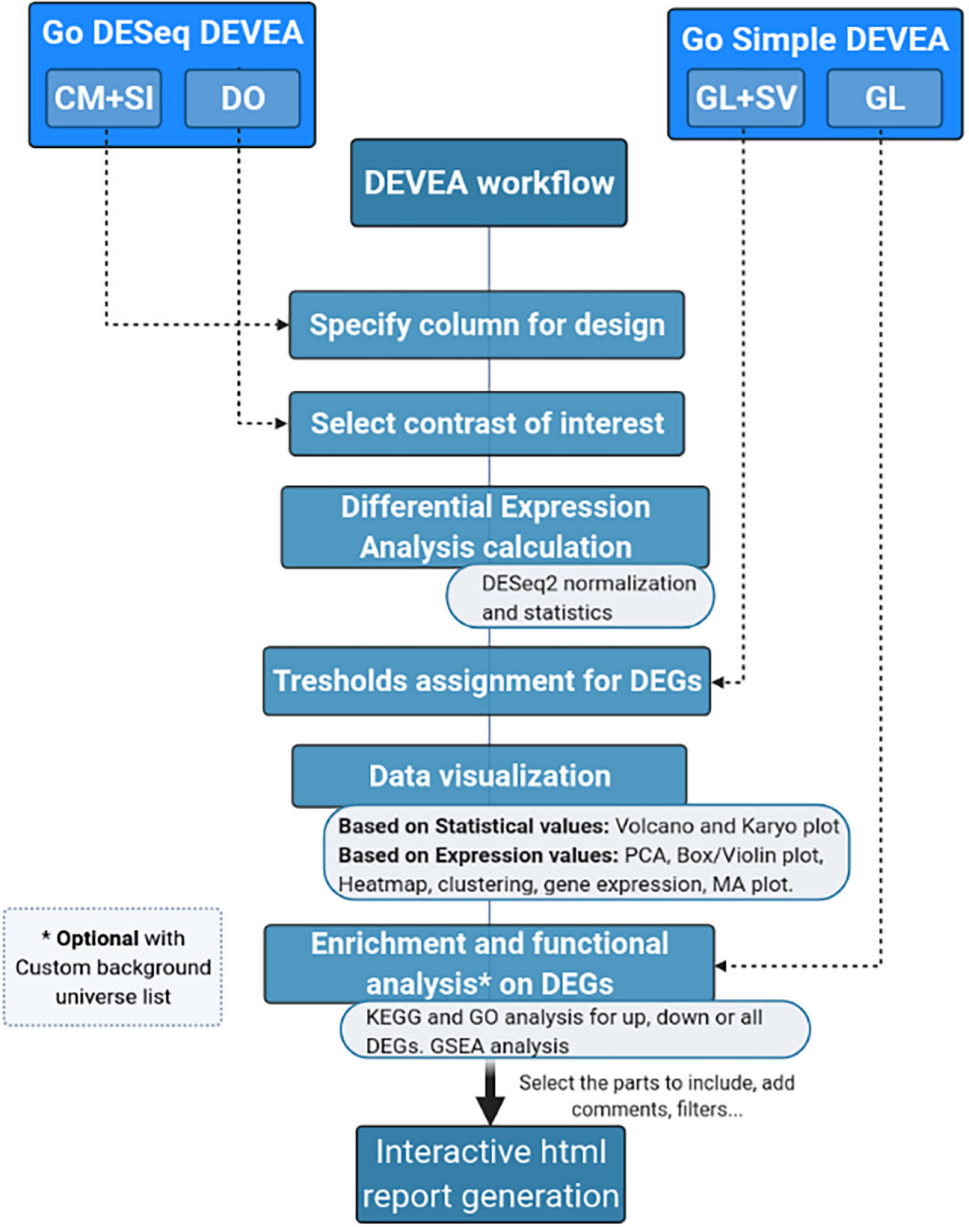
DEVEA global workflow.

The full DEVEA global workflow is shown in Figure 1. The main analysis path can be launched at different steps depending on the four input modes (check *Data requirement section* for details), represented at the top of the figure. The dashed arrows indicate where every input is incorporated in the pipeline, until the end of the workflow. Objects that are more complex will generate more results. For each step of the analysis, intermediate results are available as tables and graphical representations in their dedicated spaces, detailed below in the circular notes. The vast majority of tables and plots are interactive, allowing the user to visualize data in real-time as well as to interact efficiently and can be individually downloaded. In the end, a global report can be generated and annotated. Each of these steps of the complete analysis will be described in their corresponding sections in the next paragraphs.

### Data requirement

**Figure 2.**
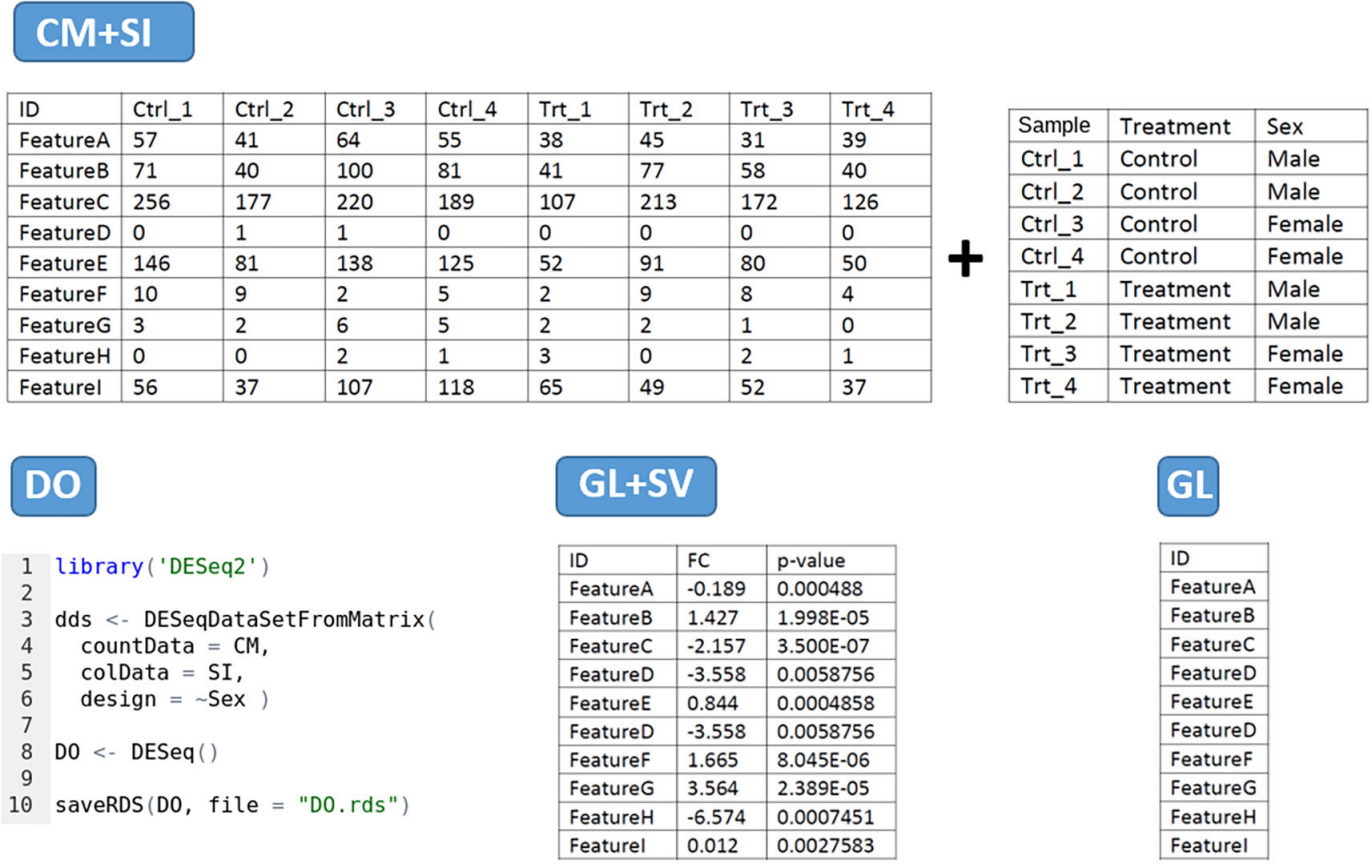
Possible data input formats for DEVEA. ‘Feature’ represents a Gene Symbol or ENSEMBL gene ID.

The tool has four main data input modes ( Figure 2):

• **CM + SI mode:** refers to a **counting matrix (CM)**, containing the raw number of counts per gene as round digits, where columns correspond to samples and rows to features. Only un-normalized raw counts are accepted as input data, as obtained from the counting process. Data from other sources containing decimals (e.g. RSEM) has to be rounded before being uploaded in DEVEA. The CM should be associated with another file gathering the **sample information (SI)**, as a data frame containing metadata about each sample, with the first column the identifier used by default as a label in visualizations. This can be modified afterward during the analysis. It should include any other relevant experimental factor (e.g. treatment/control, sex, cell type, tissue, etc). The design of the comparison will be determined by one of these factors. The column names in the CM and the first row names in the SI must be identical, and the gene IDs in this file can be included in Symbol or ENSEMBL format. Both files can be in .CSV, .TXT or .XLSX format.
• **DO mode:** based on a **DeseqDataSet object (DO)** generated by the *DESeq()* function from DESeq2 package. It is an object used to store the input values, intermediate calculations and results from a DEA. The user must have created it with the *CountData* field as the data matrix of counts, the *ColData* field with the sample information and a design formula specifying the experimental level to test for DEA. The first column in the CountData and the first row in the ColData are equal. The gene names can be included as gene Symbols or ENSEMBL gene IDs. The object must be compressed and extracted from R in .RDS format. If the differential expression object has been generated with a different tool or package, you may use the DEFormats ^19^ R function for a possible conversion.
• **GL + SV mode:** a **gene list (GL)** with associated **statistical values (SV)** per gene. The first column should contain gene names (in Symbol or ENSEMBL format), the second column the fold-change and the third column the statistical adjusted p-value, in this precise order. Column names should be provided without special characters. The numerical values will be used without further modifications by DEVEA to set the threshold of expression change and the significance. This table can be uploaded in .CSV, .TXT or .XLSX file format.
• **GL mode:** a **Gene list (GL)** consisting on a single column file in .CSV, .TXT or .XLSX format containing the gene names (in Symbol or ENSEMBL) and including a column name without special characters (i.e. GeneName, Genes, ID, etc). The gene list can be copied and pasted directly into the dedicated field in DEVEA, without the column name.

While the main data type for DEVEA’s usage is RNA-seq data, it is worth noting that the simple gene list (GL) can be built from any other type of “omics” datasets, as long as the identifiers are recognized by DEVEA. Another example could be the use of GL + SV mode with treated data from microarray analysis, where values such as FC of expression between groups and adjusted p-value are available from the signal intensity of the probes. It is highly recommended to work with log2FC and adjusted p-values. The more elaborated input data types, such as the counting matrix (CM + SI), can be built in some cases from different data where they can safely be processed by DESeq2 functions. An example may be mass spectrometry data, the method of choice for quantitative. With label-free proteomics, it is possible to quantify proteins by using their spectral counts as an approximation of protein abundance, and then use statistical models such as DESeq2 even if they are designed specifically for count data. A study comparing different statistical methods for differential expression detection in label-free mass spectrometry proteomics shows that DESeq2 performed well both in terms of detection of true positives as well as controlling for the number of false negatives. ^20^ Therefore, it is perfectly possible to use this type of data in DEVEA, as long as the values represent unique measurements as integer numbers, and the protein IDs are replaced by their coding gene name.

### Implementation

***Getting started***

**Figure 3.**
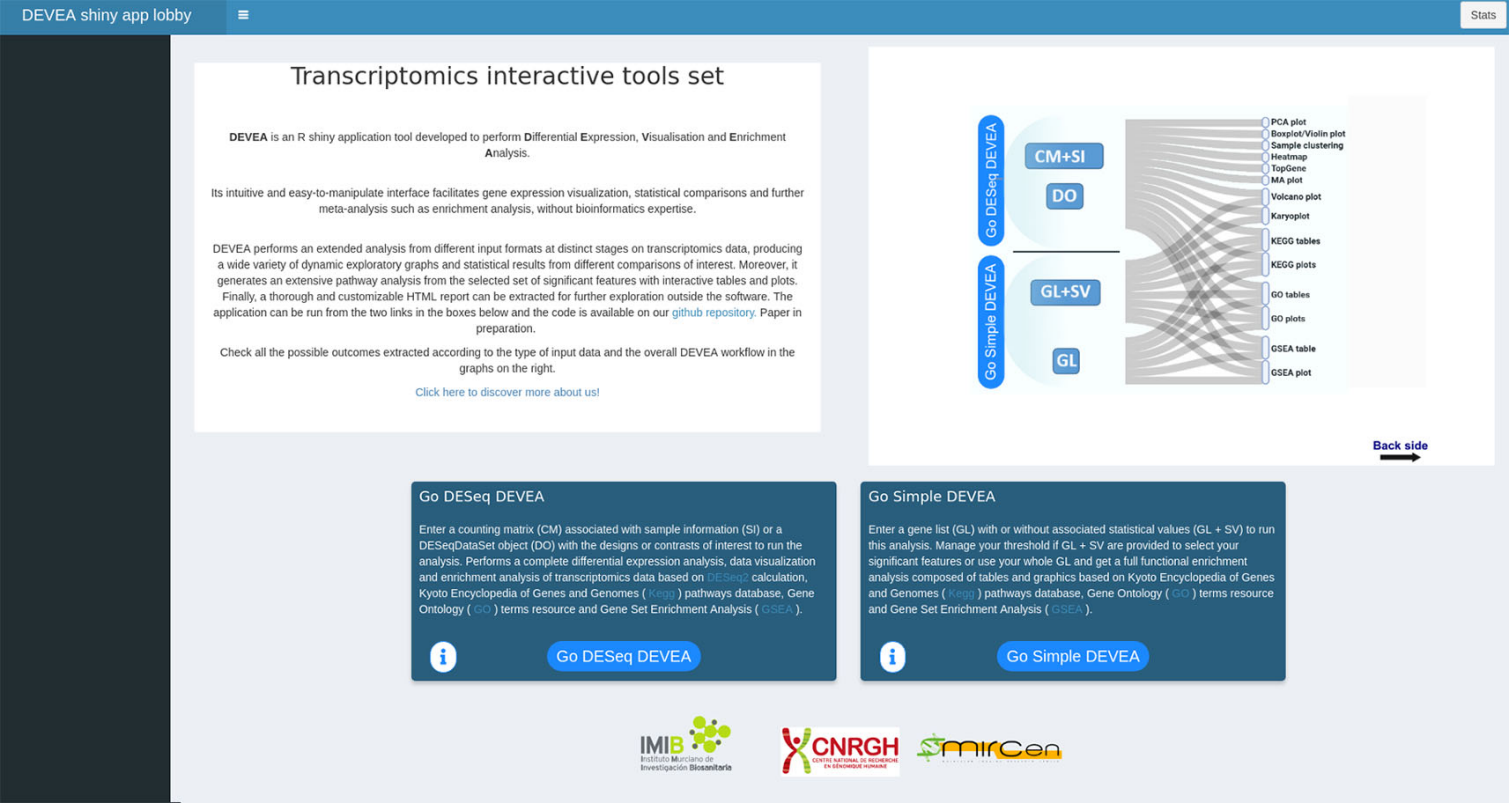
Generic screenshot of the lobby app showing the two possible pathways to start the analysis.

To start working with DEVEA, the adequate module to perform the analysis has to be chosen from DEVEA’s main lobby interface. The decision depends on the input data format. The user has to choose ‘Go DESeq DEVEA’ if their input is a CM + SI or a DO, and the ‘Go Simple DEVEA’ mode in the case of a GL + SV or a simple GL input files. See Figure 3 for a visual screenshot of the lobby.

***Data upload and statistical design specification***

**Figure 4.**
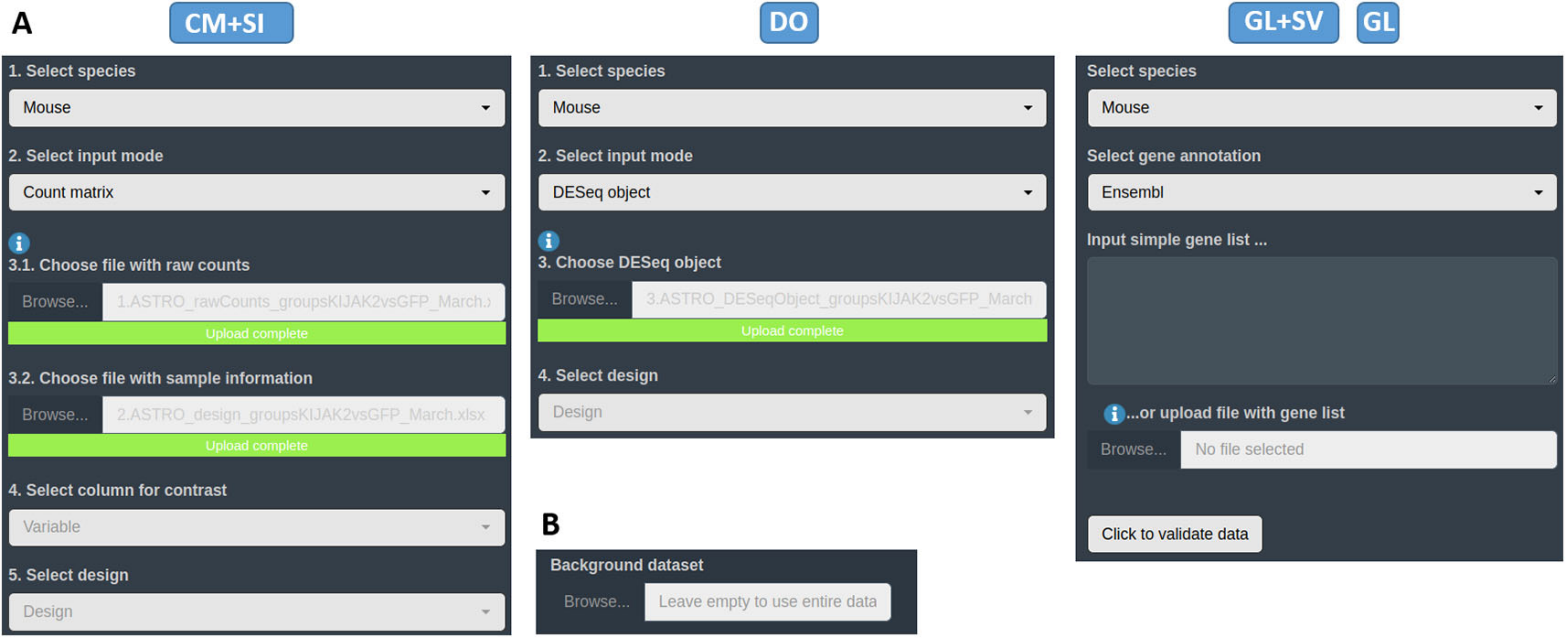
DEVEA’s import section. A: Upload spaces for different types of input data. B: Section to specify a custom background universe for enrichment analysis.

Within the appropriate interface according to the data type, the first tab available corresponds to the *Input data section.* The user has to upload their own data in one of the different accepted formats and types (see them on *Data requirement section*) in their dedicated spaces (see Figure 4A). For all input data, a field to specify the custom dataset to use as a background universe is available as well (see Figure 4B). If necessary for the user, some *demo data* representing the four different input types, can be found on DEVEA’s GitHub https://github.com/MiriamRiquelmeP/DEVEA/tree/main/data. The nature of the *demo data* and how it was generated are described in the *Use case section.*

When a CM + SI or a DO is used as input data, it is important to indicate the statistical design or contrast for the expected comparison. The design formula expresses the variables that will be used to calculate the differential expression in following steps. For the CM + SI input format, the levels of interest that will compose the final design must be included in one of the columns of SI file. By entering the column name in the ‘Select column for contrast’ field, the program extracts the conditions and calculates the combination based on the distinct levels (i.e. *Treatment_Control_vs_Treatment* if column Treatment is selected or *Sex_Male_vs_Female* will be displayed if Sex is selected from SI in Figure 2 - CM + SI). In cases where more than two levels are available, the application will propose all one-vs-one combinations. Then, the relevant combination for the analysis has to be selected by the user in the ‘Select design’ part. In a DO, the user can use the function *relevel()* from DESeq2 in R to specify the basal level.

With a DO data type, the column for the design must have been already incorporated when generating the DESeq2 object in R. Only simple designs will be generated and/or can be selected from ‘Select design’ field (i.e. *Sex_Male_vs_Female* if *design = Sex* is specified in the formula as in Figure 2 - DO). Several conditions to be treated can be included in the design of DESeq2 within the same DO. DEVEA will offer the possibility to consider any of them for each analysis, and the user can select which contrast to explore from the same DO.

It is possible to model batch effects in DEVEA with the DO object. In that case, the user can include the batch effect directly in the design formula (i.e. *design = ~ Batch + Condition*). Once the final contrast is specified as *Condition_level1_vs_level2*, the batch effect will be accounted for in the statistical model. In that case, the batch effect is treated in DEVEA as a covariate in the regression model.

The user can also remove the batch effect before uploading the DO object into DEVEA, for instance by using the *removeBatchEffect()* function of the Limma-Voom R package, ^16^ or other packages such as ComBat-seq. ^21^

***Differential expression analysis (DEA) and data view***

The first key performance of the application consists in extracting the descriptive information based on the feature expression and the statistical contrast for DEA. In CM + SI data type a new DeseqDataSet object is calculated from the files and information provided by the user. In DO or GL + SI input modes, the application retrieves the important values already included in these objects. All transformations, normalization and measurements applied to the data at this step are performed with functions included in DESeq2 R package. It should be noted that DEA calculation is not possible with the simple GL input mode, due to the lack of expression values and statistical details. The following comments will not apply to this object.

**Figure 5.**
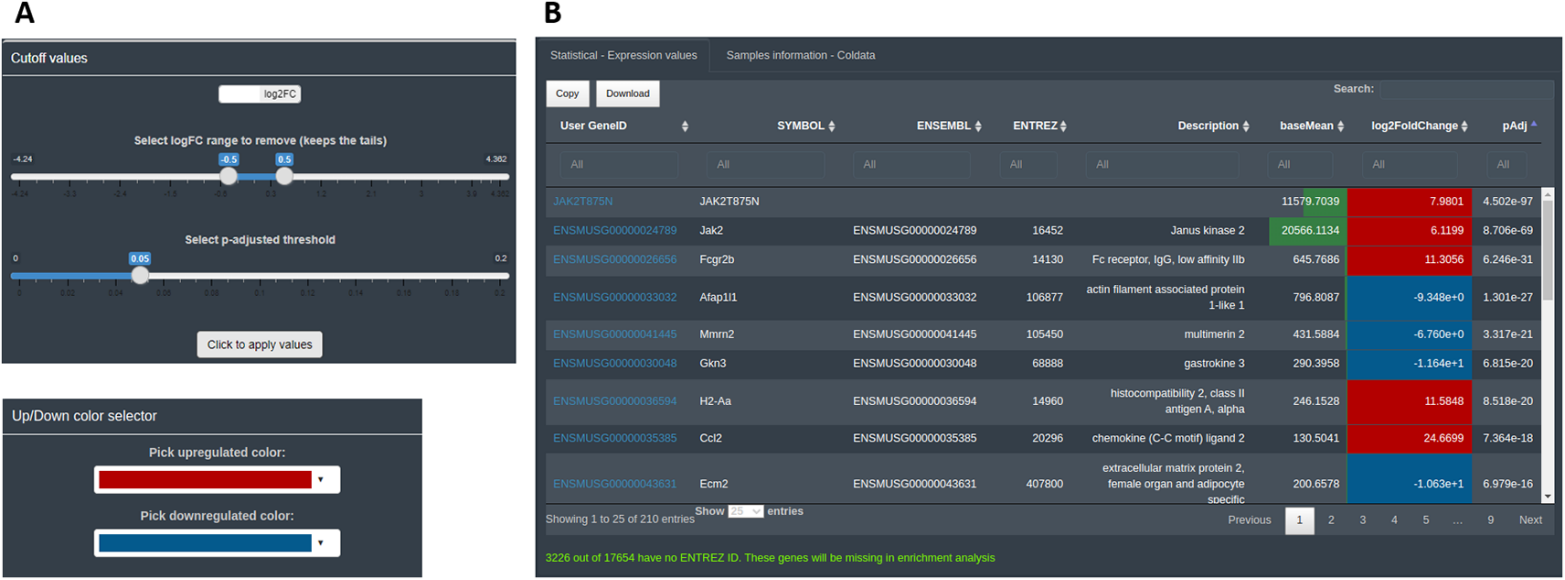
Basic data visualization for DEGs in DEVEA. A: Controls used to set the statistical thresholds and graphical features parameters. B: Interactive result table.

The calculations and the statistical results are accessible in the ‘Preview dataset’ section tab. At this step of the analysis, the user can explore the number of features considered as differentially expressed and their direction, and establish the descriptive statistics thresholds to consider them DEGs. By default, DEVEA uses prefixed log2 fold change |lfc| > 0.5 and adjusted p-value < 0.05 thresholds, that can be adapted by the user at any moment and thus modulate the list of DEGs. Moreover, the information uploaded and the descriptive statistics will be used to establish and control some interactive parts of the plots. For example, the color of defined up-regulated or down-regulated genes can be chosen ( Figure 5A). For CM + SI and DO, a complete table of results, named ‘Statistical - Expression values’, is displayed showing useful information such as base means across samples, log2 fold changes, standard errors, raw and adjusted p-values for the specific design selected. A second table is also shown with the DEA details called ‘Samples information - Coldata’ ( Figure 5B). For the GL + SV mode, raw data are displayed in a table. The user can monitor gene name conversion, explore values interactively and sort, filter and download them at any time.

**Figure 6.**
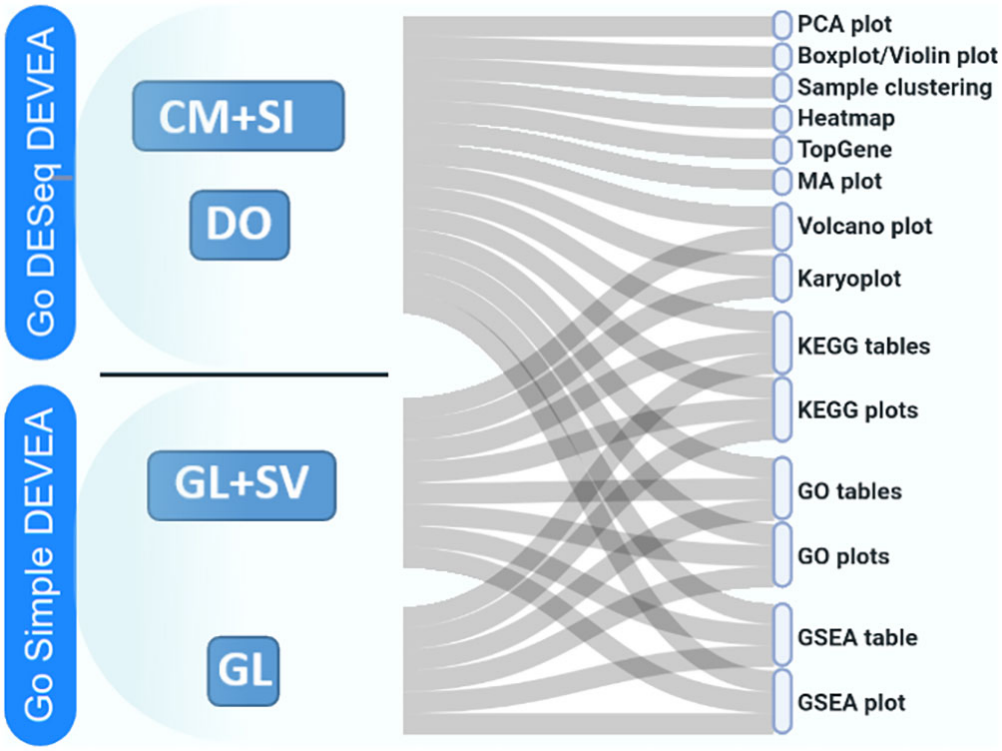
Possible types of graphical representations in DEVEA depending on data input type.

**Figure 7.**
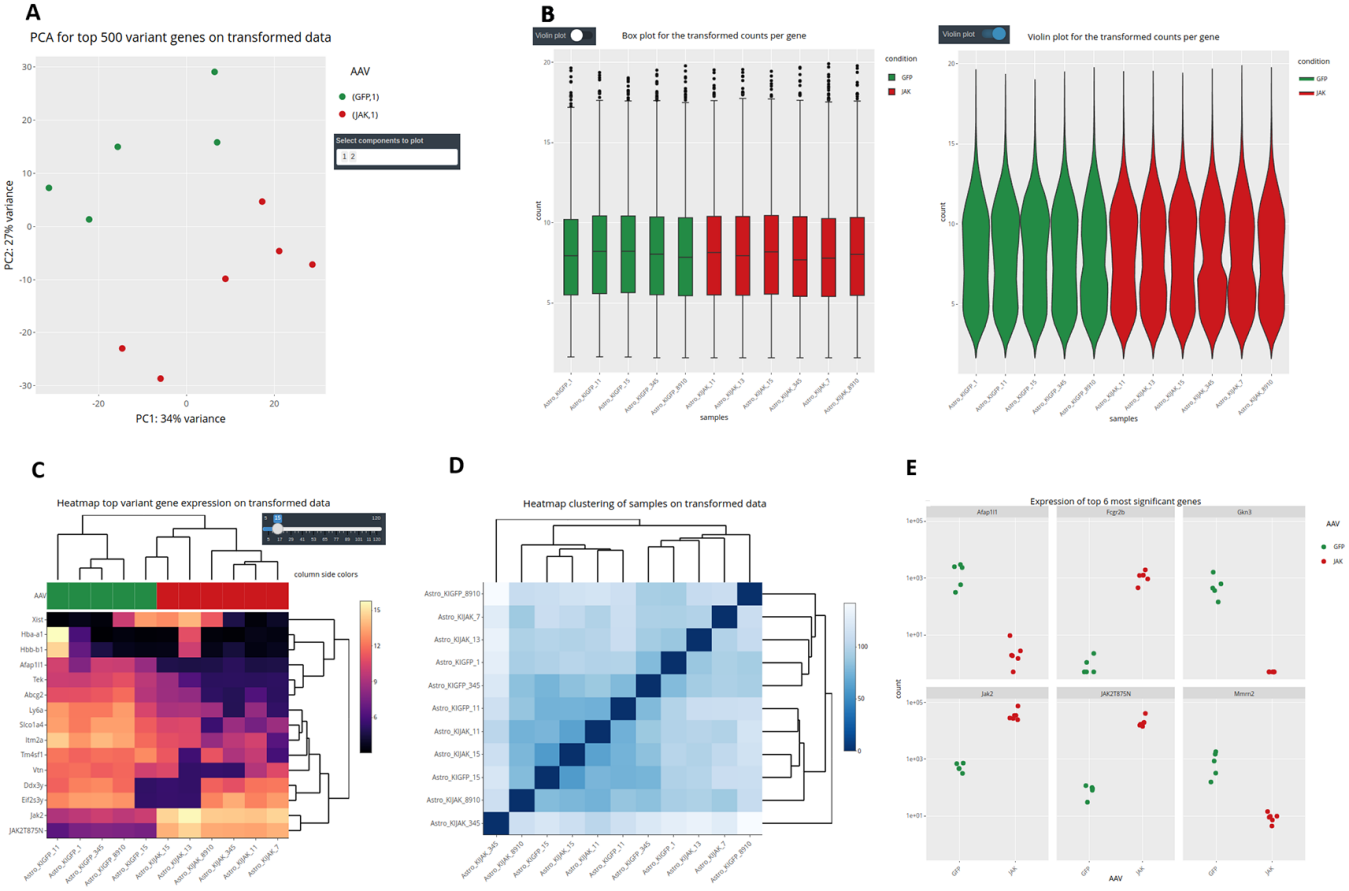
Advanced data visualization in DEVEA. A: PCA plot. B: Box and violin plots. C: Gene expression heatmap. D: Sample hierarchical clustering. E: Dot plots.

**Figure 8.**
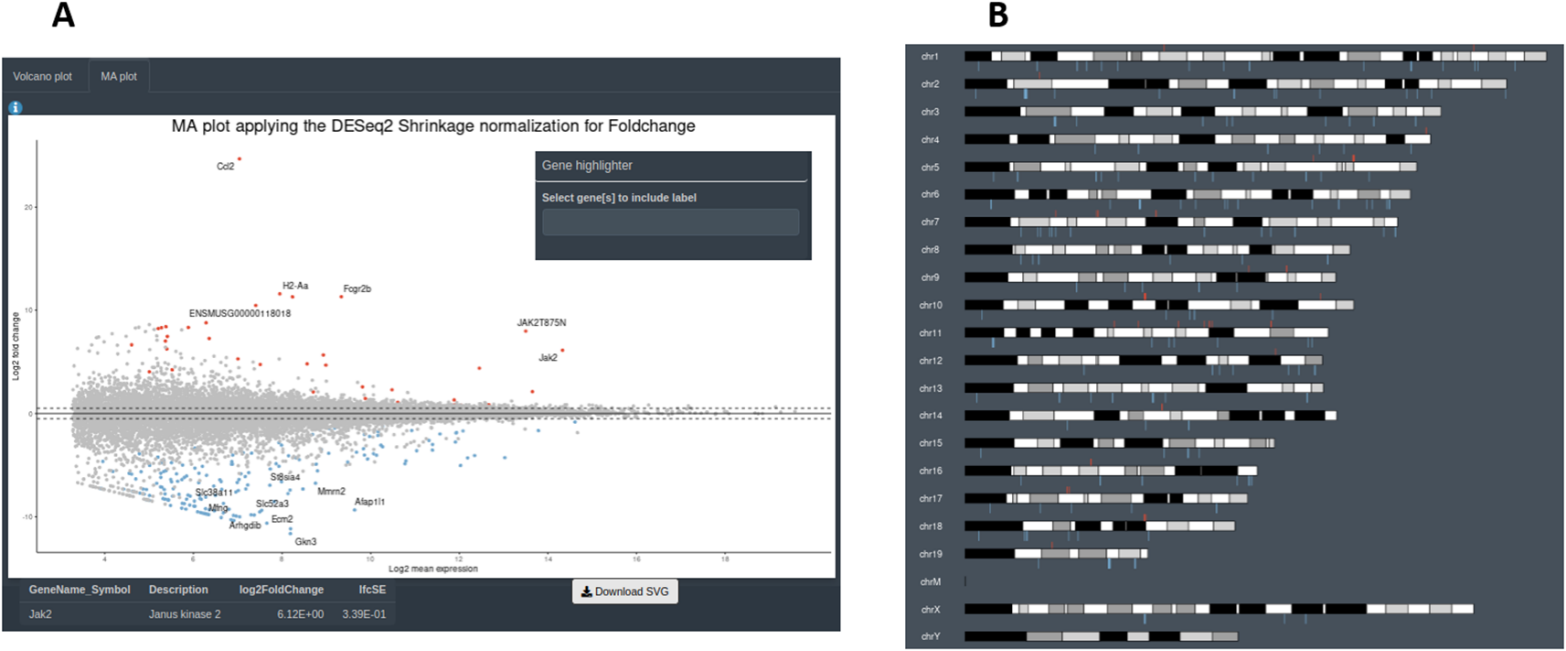
Additional data visualizations available from DEVEA. A: Section to explore volcano and MA plots. B: Karyotype plot.

It is important to stress that, as different elements can be extracted from the distinct input objects depending on their complexity, the number of graphs available for each of them vary ( Figures 1 and 6). Using the raw expression values that are available only in CM + SI and DO input modes, the user can explore data in a Principal Component Analysis plot (PCA) with the top 500 variant features, to show clusters of samples based on their similarity selecting the principal components of interest; a box or violin plot for gene expression distribution across the dataset; a heatmap representation of the top variant genes, regulated by the user; and a dot plot with the expression of the top 6 variant genes or a selection of individual genes of interest ( Figure 7). A second group of graphs represents genes related only to their statistical values. This can be displayed from CM + SI, DO and GL + SV modes. They consist in a volcano plot, that shows statistical significance (adjusted p-value) versus the magnitude of change (FC) regarding the contrast levels; and a karyotype plot showing the DEG position on the genome and the direction of change (color coded by up- or down-regulated) ( Figure 8B). Finally, it is also possible to combine gene expression with statistical values, from CM + SI and DO input modes, to generate a MA plot (an application of a Bland–Altman plot for visual representation of genomics data ^22^) ( Figure 8A) displaying feature labels and statistical values by clicking on each gene dot.

***Enrichment analysis (EA) and visualization***

The last stage of the analysis with DEVEA is EA. This is a method to identify classes of defined categories that are over-represented in the list of DEGs. These categories may be associated with disease phenotypes, biological pathways, or cellular functions. DEVEA uses the differentially expressed and significant features to retrieve the over-represented terms from several well-known databases. This major block of the DEVEA analysis can be carried out from all data input types. It consists of an extensive EA after the selection of appropriate statistical values for defining DEGs from CM + SI, DO and GL + SV, or using all components included in the simple GL input. It collects significant terms from KEGG (Kyoto Encyclopedia of Genes and Genomes ^23^) and GO (Gene Ontology ^24^) Biological process, Molecular function and Cellular component databases. Furthermore, a GSEA (Gene Set Enrichment Analysis ^25^) and leading edge exploration analysis can be performed from different databases for the whole set of features. In the case of CM + SI, DO and GL + SV input modes, KEGG and GO analyses are performed for all DEGs together, and for the subset of up- and down-regulated genes in separated tabs. GSEA is always performed on the complete set of genes and uses the statistical values associated with the features. With the simple GL data input, enrichment can only be performed for the whole set of genes for KEGG and GO analysis and no GSEA will be possible, due to the lack of statistical information.

**Figure 9.**
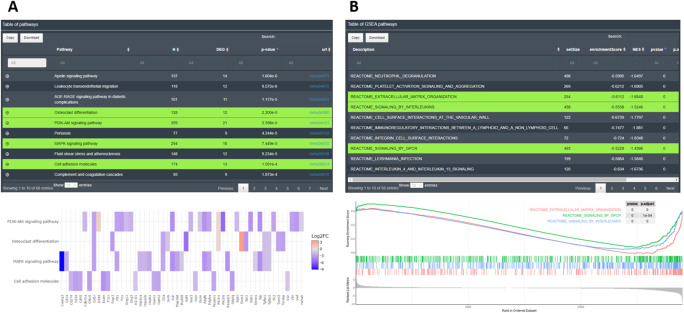
Examples of enrichment analysis with DEVEA. A: KEGG EA table and heatmap. B: GSEA table and plot.

The main results of EA are shown as interactive tables containing detailed information on the enrichment from each database. In KEGG and GO categories, the tables display columns for the name of the significant pathways or terms, their adjusted p-values and additional descriptive information such as total number of genes associated or the DEGs participating in the pathway. The user can also display the gene names that match in the pathway from the “+” symbol. Below each table, additional plots can be created by selecting rows of interest (showed in green on the Figure 9A). The plots are interactive and reactive. They can be changed at any time by selecting new lines in the table. The user can visualize results as word cloud, circle plot, bars plot, chord plot, dot plot, heatmap or net plot representing different elements from the tables. For GSEA, the results are displayed as a table containing the significant enriched pathways from the selected databases. In the table, important GSEA calculation parameters are available, as leading edge analysis. This allows determining which subsets of genes contributed the most to the enrichment signal of a given pathway. Below, a typical GSEA plot is shown from the lines selected in the table (see Figure 9B). Leading edge results have also been implemented in the plot when one unique pathway is displayed, as a red line indicating the extent of what we consider the leading edge genes.

As a special feature offered by DEVEA, a custom gene list can be uploaded to be used as the background gene universe for EA ( Figure 4B). For example, the user can use a background list containing only expressed genes in this experiment. It will control for experimental context and enable representative functions, rather than only the functions explaining the nature of the samples, to be identified. This is especially important to consider in microarray studies when a limited number of probes is used, or in studies with specific cells or tissues showing a restricted set of detectable genes.

For this EA, internal ENTREZ ID gene code is used to associate gene names in Symbol or ENSEMBL with the enrichment annotation in KEGG, GO and GSEA libraries. An exhaustive conversion across different functions is conducted to retrieve all possible terms, since they are not homogenously registered in all conversion databases. Furthermore, not all genes are curated or annotated and therefore some will be left out of the EA (the portion of genes is indicated in the application below the ‘preview table’ as shown in Figure 5B). This is a limitation from the automated databases conversions without manual curation. For this reason, the number of species is limited and not all genes will be used for the EA, but the results obtained are more robust.

***Global report***

**Figure 10.**
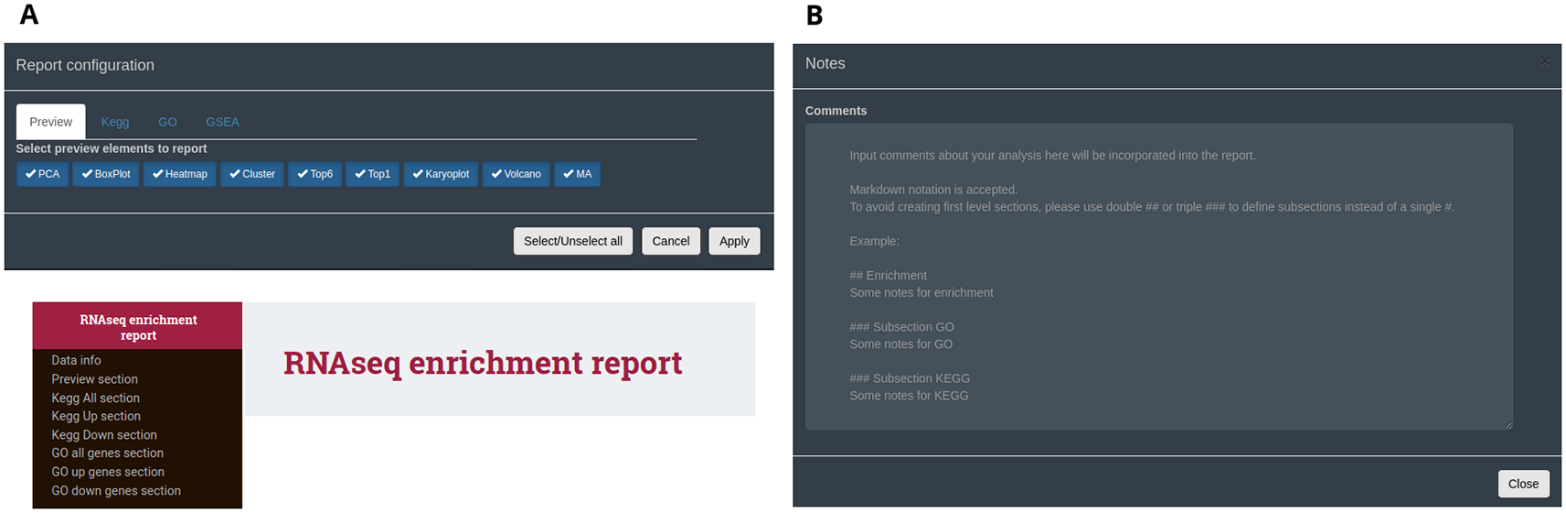
Interactive final DEVEA report functionalities and features. A: Configuring box and final HTML report. B: Notes transferable to the report.

An interactive HTML report can be generated from all data types following analysis and exploration with DEVEA. It is available in the ‘HTML report’ button at the top fixed part of the application. To create it, the user can select the individual set of figures, tables and results to be kept in this single HTML document ( Figure 10A). Plots will retain the last aesthetic indicated in the graphical parameters (e.g. colors, shapes, labels, terms). Only full tables will be kept, without taking into account potential filters applied during the analysis, allowing full exploration, sorting and re-filtering of the whole dataset outside of DEVEA. It should be noted that the majority of the results can also be copied or downloaded in high-quality format at any step of the analysis within DEVEA. Importantly, comments can be included at any step of the analysis in a dedicated section at the top right position of the application, and will be automatically saved ( Figure 10B). They can be displayed in the final report to ensure that the observations made throughout the analysis, with special interpretations or results are maintained.

Please note that if any of the graphs or tables fails to be generated in the application, when there are not enough results, genes or pathways to display them, the report cannot be generated. Unselect the problematic plots or tables to be able to render the rest of the report without errors.

## Use case

**Figure 11.**
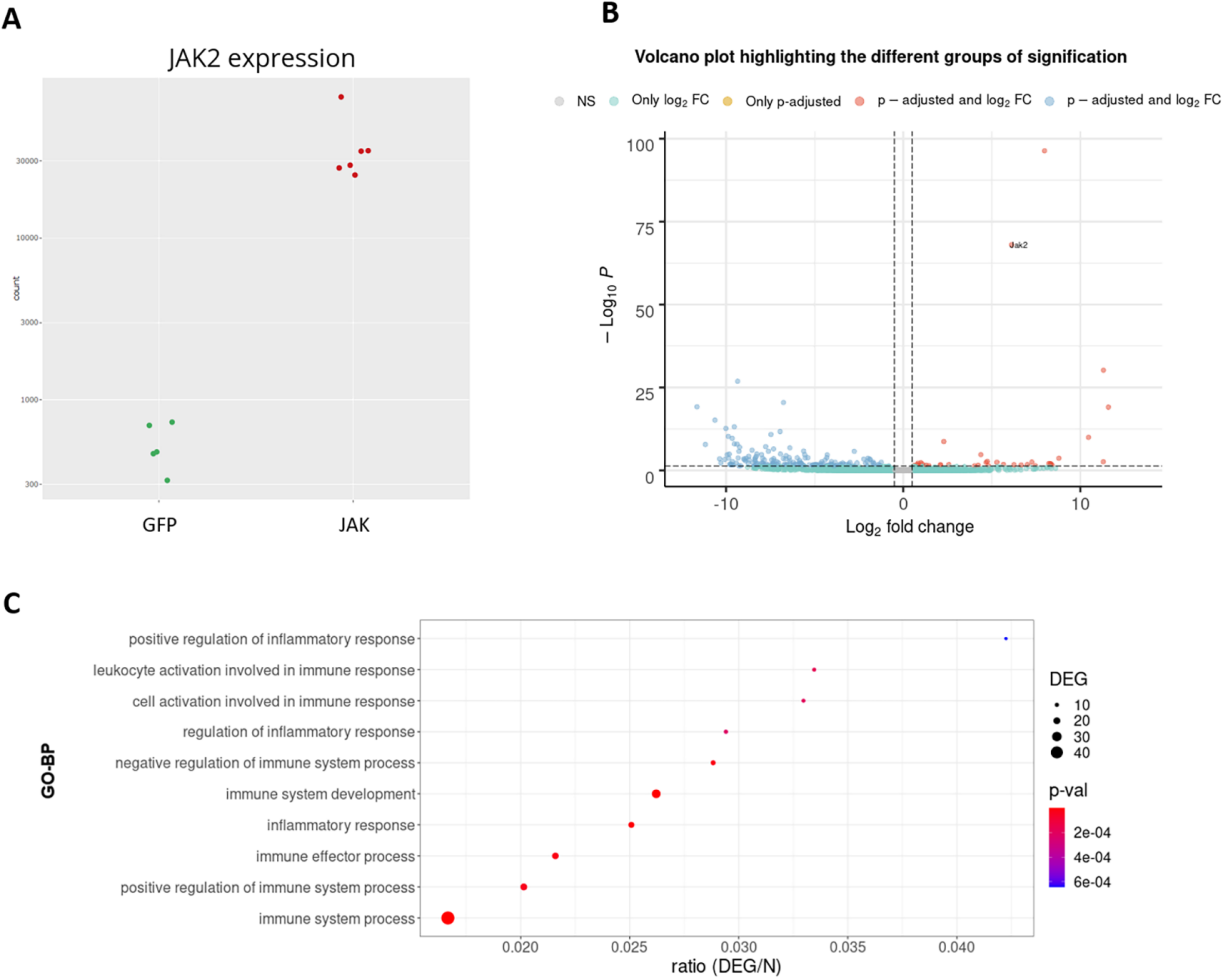
RNA-seq analysis of the JAK2-STAT3 pathway in astrocytes of a Huntington’s disease model mouse. A: Expression levels of the *Jak2* gene between groups. B: Volcano plot. C: GO-BP enriched categories.

To demonstrate the usefulness of DEVEA, an RNA-seq dataset generated by our team and published recently ^26^ was investigated using the application. The study aimed to characterize the role of JAK2-STAT3 signaling in astrocytes in the context of Huntington’s disease (HD). HD is a rare genetic neurodegenerative disease leading to severe motor, cognitive and psychiatric symptoms, with no curative treatment available. ^27^ Astrocytes, a heterogeneous group of star-shaped glial cells, perform key functions in the brain. They provide nutrients to neurons, regulate synaptic transmission, and contribute to brain repair following injury. ^28^ Astrocytes become reactive in the brain of HD patients and their impact on HD progression is still unclear. ^29^ The study used a genetic mouse model of HD. Treated mice were injected with an adeno-associated viral (AAV) vector targeting astrocytes and encoding a constitutive form of the JAK2 kinase (JAK2T875N) to activate the JAK2-STAT3 pathway. Control mice were injected with a similar AAV expressing GFP. Astrocytes were isolated and sequenced by RNA-seq on a HiSeq 2500 Illumina platform (2 × 100 bp). Quality control of sequencing data was performed with FastQC ^30^ (v0.11.9). Reads were mapped on the GRCm38 (mm10) mouse genome assembly with Hisat2 ^31^ (v2.2.1), and a counting matrix was generated. Quantification of reads associated with genes was achieved with featureCounts ^32^ (v2.0.0), and differential gene expression analysis was performed with DESeq2 (v1.28.1) Bioconductor (v3.13) package on R (v4.0.2). Only genes with a raw number of counts ≥ 10, in at least 3 samples were analyzed. Data were adapted and integrated as different input objects in DEVEA to test all functionalities. For instance, Figure 7A shows that control (GFP, N = 5, in green) and treated (JAK, N = 6, in red) samples are clearly separated on a PCA plot, with a better separation achieved on PC2 (representing 27% of the total variance). Figure 7C- D also show two types of clustering profiles, which group samples from the same group together and display genes with higher variability. Figure 11A shows that the levels of *Jak2* are higher in treated (JAK) versus control (GFP) groups, as expected by AAV-mediated gene transfer (log2(FC) = 6, associated adjusted p-value 8.7E-69). In addition, a volcano plot demonstrates that JAK2 causes down-regulation of many genes, shown in blue in the upper left quartile of the graph on Figure 11B. Finally, EA in the treated (JAK) versus control (GFP) list of DEGs shows that many GO-BP terms are related to Immunity/Inflammation, a process linked to the reactive changes in astrocytes induced by JAK2 ( Figure 11C).

All data corresponding to this research project are available under the four different DEVEA input types. They are available on the GitHub web site (see the *Software Availability section*). They consist in (1) a set of CM + SI, where features are genes in the ENSEMBL format and expression represents raw counts after alignment and filtering to remove non-expressed genes from the CM. The final number of features is 18,260 (+ 2 custom genes representing the two transgenes *Jak2T875N* and *Gfp*). The SI file contains all relevant information on sample characteristics; (2) a complete DO built from the same CM and SI files with a design based on the comparison of the two different AAVs (design = AAV); (3) a GL + SV file manually created according to the *DESeq2* results. The file is generated with 3 columns that contains the gene names, FC and p-values for the top 500 most significant genes and (4) a unique GL containing only the 268 DEGs from the comparison of interest, reported from the DESeq2 analysis with adjusted p-value < 0.1 statistical threshold and no threshold for the FC (for further details on the input data, see the *Data requirement section*).

With this user case, no errors were detected throughout the analysis. DEVEA, thanks to a large range of graphical and statistical analyses, highlighted significant differences between reactive astrocytes in the JAK group and control astrocytes in the GFP group. Due to extensive EA, DEGs were associated with important biological functions such as immunity/inflammation pathways as well as cytokine signaling and proteostasis. These results are consistent with the conclusion described in the corresponding publication. ^26^

We have also generated demo files corresponding to an RNA-seq study of *Arabidopsis thaliana* from a recent publication. The data represents the differential expression analysis of 3 mutant fibrillins (FBN6) samples vs 3 controls. See the dedicated paper for further details. ^33^

Apart from these demo data, to facilitate the preparation of some input data for biologists who lack bioinformatics skills, we have provided on our GitHub two standard tutorials using the Galaxy platform. ^34^ The tutorials allow a user with no prior bioinformatics knowledge to generate read counts from raw RNA-seq .fastq data in two easily accessible step-by-step methods. Quality control and a few downstream analyses similar to those done within DEVEA can be explored following this pipeline. The tutorials can be accessed at https://github.com/MiriamRiquelmeP/DEVEA/blob/main/Galaxy_tutorials.md.

## Related works (state-of-the-art)

The field of application tools for transcriptomics data visualization, DEA and EA is constantly growing. DEVEA functionalities are compared with six similar applications recently published (iDEP, ^5^ ShinyGO, ^35^ DEGenR, ^36^ GENAVi, ^6^ RNfuzzyApp ^37^ and ideal ^7^). These tools operate through a graphical user interface, provide interactive results, and are based on stable and maintained R packages. A detailed comparison of the DEVEA main attributes is shown in Table 1. Characteristics that are related to data management and importation of data into the application have been stressed in the upper part of the table, followed by the various modes of DEG identification and different interactive graphical results, EA calculations and global reporting and webhosting. It is clear from Table 1 that most of the selected applications share many functionalities with DEVEA. One exception is the application ShinyGO, which offers significantly fewer options since it is designed only to perform enrichment calculations from simple gene lists. However, some other specific differences exist with other tools. DEVEA has more flexibility in terms of data type import in different formats, representing different stages of the analysis. Similarly, the user has a wide range of exploration possibilities via GL and GL + SV input formats, in terms of data generation and origin. The list of features and the associated statistics may have been generated from many different external tools or could represent several analysis types, as long as they are eventually converted into gene names (i.e. Microarray data results, proteomics results, gene lists from the literature, etc.). As a further advantage, the ability to import complex objects, such as DO, increases the number of visualization and analysis options. Despite this, not all possible visuals that can be generated from these objects are included in DEVEA, which has room for improvement. In particular, DEVEA could further develop data management functionalities, by extending the capacity of dealing with batch effects or data pre-processing and filtering. One of the possible drawbacks is the low number of available species for the EA compared with other tools or the potential mismatches when converting gene names. The EA, where there is information about the level of expression, can be generated from all DEGs, either split by the direction of expression change (only on up- or down-regulated genes), or merged into a single list. This is not always the case in applications that perform analyses on the whole list of potential genes of interest. The custom global report, a unique feature of DEVEA, might be very handy to share results with collaborators, because the user can easily insert comments and transfer the fully-interactive HTML report. Lastly, DEVEA appears slightly more flexible in terms of application hosting and running, since it is possible to run it online (DEVEA web server), or offline using R. For instance, certain applications such as DEGenR and RNfuzzyApp do not offer the possibility to run the application online.

**Table 1.** DEVEA functionalities compared to similar software tools in their online versions.

Publication year	DEVEA	iDEP ^5^	ideal ^7^	GENAVi ^6^	RNfuzzyApp ^37^	DEGenR ^36^	ShinyGO ^35^
	2022	2018	2020	2019	2021	2021	2020
**IMPORT DATA/MANAGEMENT:**							
**Count data input mode**	x	x	x	x	x	x	
**DESeq object input mode**	x						
**Gene list input mode**	x						x
**Several gene names**	x	x	x	x	x	x	x
**>2 species**	x	x	x		x		x
**Raw data accessibility**	x	x	x	x	x		
**Demo data**	x	x	x	x			x
**DEA COMPUTATION & DATA VISUALIZATION:**							
**DEA statistical calculation**	x	x	x	x	x	x	
**Manage stats threshold**	x	x	x	x		x	
**Interactive statistics summary table**	x		x	x	x	x	
**Interactive preview visuals**	x	x	x	x	x	x	
**Interactive DE visuals**	x	x	x	x	x	x	
**ENRICHMENT ANALYSIS & VISUALIZATION:**							
**Custom background universe**	x						x
**Split results by DE directions**	x		x		x		
**KEGG results**	x	x		x	x	x	x
**GO results**	x	x	x	x	x	x	x
**GSEA type results**	x	x	x			x	
**Interactive EA tables**	x	x	x	x	x	x	x
**Interactive EA visuals**	x	x	x	x	x	x	x
**DOWNLOAD & REPORT:**							
**Individual plots download (.SVG, .HTML, .PNG)**	x	x	x	x	x	x	x
**Interactive report**	x		x	x			
**Custom report**	x						
**ACCESSIBILITY:**							
**Public server**	x	x	x	x			x
**Source code available**	x	x	x	x	x	x	x

Although overall applications share common analysis blocks, DEVEA presents more graphical variety than most of them. For example, as a criterion to consider in the table that an application contains “Interactive preview visuals” to preliminary explore the data, only one of PCA plot, violin/box plot of sample profile, heatmap for gene expression, sample clustering, gene expression dots plot per groups should be generated from the applications. For “Interactive DE visuals” of the DEA statistics and profile the criterion consist of including at least one of volcano plot, MA plot or karyoplot. Finally, for the “Interactive visuals” to navigate the EA, the marked tools include at least one plot among bars plot, dots plot, chord plot, heatmap, net plot, word cloud, circle plot of the enriched terms. For most applications, only a small subset of these plots are implemented. DEVEA contains all these graphs, requirement that none of the others met.

## Conclusion and Future Perspectives

The DEVEA application is developed to improve the set of existing software to perform DEA, data visualization and annotation or EA from transcriptomics data. DEVEA meets the need for applications that give sufficient usage autonomy, without compromising the complexity and accuracy of the results. It provides an interactive and user-friendly interface accessible to users without bioinformatics training, with a high diversity of analysis components. Researchers can explore their data in real-time, carry out DEA and subsequent EA from distinct well-known databases without losing possible customization. DEVEA contains a large range of functions in a single tool, avoiding the use of different tools/websites to perform transcriptomics analysis, offering advanced ready-to-publish visuals, tables and results. One of the main strength is the incorporation of several input data types. Additionally, the use of robust R packages, especially the DEA DESeq2 package is an attractive functionality of the application. The possibility to include a custom background makes DEVEA better suited for analysis in which correction of some experimental bias could lead to better results in the EA, avoiding the inappropriate inflation of statistical p-values and false-positive results. Another key advantage is that DEVEA allows the user to extract their results individually or in an interactive format through a custom HTML format file. To further develop DEVEA analyses, we plan to offer additional pre-treatment options (e.g. removing batch effect, filtering genes by expression, etc.) and integrate more species and include transcription factor enrichment analysis. In conclusion, the purpose of DEVEA is to promote the dialogue between biologists and bioinformaticians, particularly to produce suitable data and to understand the validity of the data needed to create the best downstream results.

## Software availability

• Software available at http://shiny.imib.es/devea/. Archived source code as at time of publication: https://doi.org/10.5281/zenodo.6657245.
• Latest source code on https://github.com/MiriamRiquelmeP/DEVEA.
• Test files for every input mode can be found also on https://github.com/MiriamRiquelmeP/DEVEA/tree/main/data.
• Tutorial accessible from both DEVEA modules (DESeq DEVEA and Simple DEVEA) in the ‘Tutorial’ section from the top controls and independently on https://shiny.imib.es/DESeqDevea/tutorial.html or https://shiny.imib.es/simpleDevea/tutorial.html.

License: Apache license 2.0.

## Author contributions

MRP and FPS conceived the application, did the development and wrote the manuscript. CE, SB and EB supervised the work, tested the application and wrote the manuscript. JFD contributed fund acquisition and resources for the project. All authors discussed the results and contributed to the final manuscript.
