# Supplementary material for: DEVEA: an interactive shiny application for Differential Expression analysis, data Visualization and Enrichment Analysis of transcriptomics data
Source: F1000Res. 2023 Mar 24;11:711. Originally published 2022 Jun 28. [Version 2] doi: 10.12688/f1000research.122949.2 (PMC10043628; doi:10.12688/f1000research.122949.2)
Supplement: Peer review history

# Peer review: Reviewer response for version 2

**DOI:** 10.5256/f1000research.144696.r167705

**Published:** 2023-03-27

**Authors:** Wenbin Guo

**Affiliations:** 1 Information and Computational Sciences, James Hutton Institute, Dundee, UK

In response to the feedback provided in the last review, the authors have carefully reviewed and revised their manuscript, taking into account all the comments and suggestions made. They have added new information where necessary to further support their claims. The authors have made sure that their arguments and findings are presented clearly and logically. Overall, the authors have shown great attention to detail and have made improvements to their manuscript.

Are the conclusions about the tool and its performance adequately supported by the findings presented in the article?

Yes

Is the rationale for developing the new software tool clearly explained?

Yes

Is the description of the software tool technically sound?

Yes

Are sufficient details of the code, methods and analysis (if applicable) provided to allow replication of the software development and its use by others?

Yes

Is sufficient information provided to allow interpretation of the expected output datasets and any results generated using the tool?

Yes

Reviewer Expertise:

Transcriptomics method development, automated pipeline development, Shiny App development, bioinformatics analysis, RNA-seq data analysis, differential gene expression analysis, differential alternative splicing analysis, gene regulatory network analysis

I confirm that I have read this submission and believe that I have an appropriate level of expertise to confirm that it is of an acceptable scientific standard.

# Peer review: Reviewer response for version 1

**DOI:** 10.5256/f1000research.135003.r152235

**Published:** 2022-10-10

**Authors:** Wenbin Guo

**Affiliations:** 1 Information and Computational Sciences, James Hutton Institute, Dundee, UK

**Summary**

 The author developed a shiny App DEVEA for biologists who lack extensive bioinformatics skills to perform complex differential expression analysis and downstream function annotation and pathway enrichment analysis. It provides an easy-to-use platform and empowers biologists to hand on complex RNA-seq analysis by themselves. The multiple options of inputs, including (a) the raw read counts of genes, (b) the intermediate dataset produced by DESeq2, (c) the gene list with statistics of significance and (d) the gene list only, allowed users to start the analysis flexibly from different types of data. It also provided interactive figures and tables to visualise the results in real-time. In the end, a report can be generated with user-selected results and figures, which improved the reproducibility of the RNA-seq analysis.

 Below are some limitations of the current DEVEA and suggestions for future improvements. The author already had a plan to address some of them in future directions.

- It seems that the current version of DEVEA only supports the analysis of human, mouse and rat, which limits its usage in wider studies of plant species and other animal species.
- Due to the above limitation, the current manuscript lacks some case studies in other species, especially in plants.
- The controls for errors caused by unwanted variations are not proposed in the DEVEA pipeline. For example, RNA-seq data often have batch effects between replicates and they will largely hinder the accuracy of differential expression analysis. DESeq2 also provides suggestions on how to correct the batch effects in the user manual.
- DEVEA only allows a pair-wise comparison between control and treatment at a time, which is not flexible for complex experimental design, such as datasets with a great number of conditions to compare, datasets of time-series data and development series, and datasets with multiple groups and each group include addition level of conditions to compare.
- It would be impressive to enable enrichment analysis for the genes lacking annotations. For example, getting the gene annotation by blasting the sequence against some databases. It will be especially useful for the analysis of newly assembled genes and transcripts.

**Comments for manuscript improvement:**

- The preparation of some input data is still difficult for biologists who lack bioinformatics skills. For example, getting the raw read counts requires bioinformatics skills to map the RNA-seq data to a reference genome or transcriptome and generate read counts by using quantification tools such as Salmon, Kallisto and RSEM. The author should propose an easy-to-access method and provide step-by-step tutorials for biologists to generate the read counts from raw RNA-seq data, for example, using the Galaxy platform.  
- There are massive methods available for the function of differential expression (DE) analysis. Many comparison studies showed that the methods DESeq2, Limma-voom, edgeR and Sleuth have similar performance in DE analysis and each of these methods has a big user group. To attract more users to DEVEA, the author could highlight the advantages to use DESeq2 in DEVEA compared with other methods, such as Limma and edgeR, in the introduction or discussion.
- In the tutorial, the table of contents is floating and overlaps with texts and videos. Is it possible to reallocate the position of the table of contents to avoid overlapping?
- In the example data link: https://github.com/MiriamRiquelmeP/DEVEA/tree/main/data, it would be better to include a readme file with descriptions of the example datasets.
- “Supplemental-Information.txt: Running DEVEA locally on a machine”: I would recommend the author build DEVEA as an R package. Then, instead of “Download the compressed folder from the DEVEA GitHub repository”, users can install and run DEVEA on a local machine as an R package.
- Page 3: “Despite the great progress to facilitate transcriptomics computational analyses, some improvements are still possible for these tools.” Although comparisons were given in the Related work section, the author should give brief descriptions of these tools and summarise their limitations as literature reviews in the introduction. The author also should address what are the possible improvements and why they are needed.
- Page 11: “Reads were aligned on the mouse genome (ENSEMBL GRCm38 release 96) and a count matrix was generated.” For reproducibility, the tool for read mapping and count matrix generation should be referred to and cited.
- Figure 7: the font size of labels and some details of the figures are too small. Would it be better to change the multiple plot layout to 3 rows x 2 columns?

The paper has quite a few typos. The author should carefully check the entire manuscript again. Here, I have picked out some of them:

- Page 3: “key steps of a complete RNA-seq analyses pipeline” should be “key steps of a complete RNA-seq analysis pipeline”
- Page 3: “detailed bellow in the circular notes” should be “detailed below in the circular notes”.
- Page 5: “the simple gene list (GL) can be build from” should be “the simple gene list (GL) can be built from”
- Page 6: “the program extract the conditions and  calculates” should be “the program extracts the conditions and  calculates”
- Page 11: “To demonstrate the usefulness of DEVEA, a RNA-seq” should be “To demonstrate the usefulness of DEVEA, an RNA-seq”
- Page 12: “which offers significantly less options since” should be “which offers significantly fewer options since”

Are the conclusions about the tool and its performance adequately supported by the findings presented in the article?

Yes

Is the rationale for developing the new software tool clearly explained?

Yes

Is the description of the software tool technically sound?

Yes

Are sufficient details of the code, methods and analysis (if applicable) provided to allow replication of the software development and its use by others?

Yes

Is sufficient information provided to allow interpretation of the expected output datasets and any results generated using the tool?

Yes

Reviewer Expertise:

Transcriptomics method development, automated pipeline development, Shiny App development, bioinformatics analysis, RNA-seq data analysis, differential gene expression analysis, differential alternative splicing analysis, gene regulatory network analysis

I confirm that I have read this submission and believe that I have an appropriate level of expertise to confirm that it is of an acceptable scientific standard, however I have significant reservations, as outlined above.

# Response

**Published:** 2023-02-20

**Authors:** Miriam Riquelme-Perez

**Affiliations:** MIRCen (CEA - Univ. Paris-Saclay, FR), France

**We thank the reviewer for taking the time to review our paper and test our application. We provide here a detailed response (in bold, after + symbol) to all the comments (introduced by -, in italic) and how we addressed the majority of them.**

*- It seems that the current version of DEVEA only supports the analysis of human, mouse and rat, which limits its usage in wider studies of plant species and other animal species.  Due to the above limitation, the current manuscript lacks some case studies in other species, especially in plants.*

**+ We acknowledge that there is limited support for plant and animal species in the current version of DEVEA. We are planning to expand the number of species available in future versions. For this version, following the reviewer's suggestion, we have now included the model plant species *Arabidopsis thaliana* in DEVEA. A demo dataset has also been created compatible with DEVEA for this species, based on an RNA-seq study published recently (Lee et al. New Phytologist, 2020, 225(4):1715-1731).**

*- The controls for errors caused by unwanted variations are not proposed in the DEVEA pipeline. For example, RNA-seq data often have batch effects between replicates and they will largely hinder the accuracy of differential expression analysis. DESeq2 also provides suggestions on how to correct the batch effects in the user manual.*

**+ We can distinguish between modeling the batch effect using DESeq2 within DEVEA and removing the batch effect before importing the data into DEVEA. An advantage of using as input data a DO generated previously in R, is that the user can include Batch in the design formula as “design = ~Batch + Condition”. Once the final contrast is specified as Condition_level1_vs_level2, the batch effect will then be 'accounted for' (the statistical inferences will be adjusted for Batch) with a differential expression analysis testing for Condition. Batch is essentially treated in DEVEA as a covariate in the regression model, just as we do in association studies. If the user wants to effectively remove the batch effect for downstream analyses, it can be performed outside DEVEA on the variance-stabilized or rlog expression values using limma::removeBatchEffect() or ComBat-seq tool, before uploading the data to the application again. This could be considered for future versions of DEVEA as well. We have added a section in the ‘Data upload and statistical design specification’ section of the manuscript.**

*- DEVEA only allows a pair-wise comparison between control and treatment at a time, which is not flexible for complex experimental design, such as datasets with a great number of conditions to compare, datasets of time-series data and development series, and datasets with multiple groups and each group include addition level of conditions to compare.*****

**+ Multiple experimental conditions can be included in the design of the DESeq2 object (DO). DEVEA will offer the possibility to consider any of them foreach analysis. For instance, when “design = ~ConditionA + ConditionB + ConditionC”, the tool will propose as possible contrast 1.ConditionA_level1_vs_level2, 2.ConditionB_level1_vs_level2 and 3.ConditionC_level1_vs_level2. The user can select from DEVEA interface, which specific contrast to explore. In addition, for DO and CM + SI, when there are more than two levels from the same condition, DEVEA will propose all the one-vs-one combinations. Therefore, if the “design = ~ ConditionWith4levels”, the possible contrasts would be 1.Condition_level1_vs_level4, 2.Condition_level2_vs_level4, and 3.Condition_level3_vs_level4, level4 being the basal condition for every contrast. For CM + SI, these levels are selected alphabetically. In a DO, the user can use the function relevel() from DESeq2 in R to specify the basal level. We have changed the text in the manuscript to indicate these possibilities, in the ‘Data upload and statistical design specification’ from the ‘Implementation’ section.**

*- It would be impressive to enable enrichment analysis for the genes lacking annotations. For example, getting the gene annotation by blasting the sequence against some databases. It will be especially useful for the analysis of newly assembled genes and transcripts.*

**+ We agree that such an initiative would provide a genuinely useful service, especially for recently sequenced organisms who do not have yet a good and comprehensive annotation. However, this task is far from being trivial and requires a substantial amount of software development and is therefore currently beyond the scope of the current DEVEA implementation. For DEVEA, we currently aim to exploit available packages and resources for visualization, differential expression analysis and enrichment from well-known and described species.**

*Comments for manuscript improvement:*

*- The preparation of some input data is still difficult for biologists who lack bioinformatics skills. For example, getting the raw read counts requires bioinformatics skills to map the RNA-seq data to a reference genome or transcriptome and generate read counts by using quantification tools such as Salmon, Kallisto and RSEM. The author should propose an easy-to-access method and provide step-by-step tutorials for biologists to generate the read counts from raw RNA-seq data, for example, using the Galaxy platform.*

**+ It is true that data preparation can be quite complicated for non-specialists. Following the remark of the reviewer, we have added references and web links for two complete pipelines and tutorials for Galaxy on our DEVEA GitHub page (https://github.com/MiriamRiquelmeP/DEVEA/blob/main/Galaxy_tutorials.md). We have also included a brief explanation of them in the ‘Use case’ section.**

*- There are massive methods available for the function of differential expression (DE) analysis. Many comparison studies showed that the methods DESeq2, Limma-voom, edgeR and Sleuth have similar performance in DE analysis and each of these methods has a big user group. To attract more users to DEVEA, the author could highlight the advantages to use DESeq2 in DEVEA compared with other methods, such as Limma and edgeR, in the introduction or discussion.*

**+ It is correct that there are several methods available for RNA-seq differential expression analysis with comparable performances. We chose DESeq2 because it is widely used, very robust and recognised in the community. As suggested by the reviewer, we now stress, in the  ‘Operation’ and briefly in the ‘Conclusion and Future Perspectives’ sections, the robustness of the method by mentioning an article that compares DESeq2's performance with other methods (Baik et al., PLoS One, 2020, 15(4):e0232271).**

****

*- In the tutorial, the table of contents is floating and overlaps with texts and videos. Is it possible to reallocate the position of the table of contents to avoid overlapping?*

**+ We have noticed that the HTML file that displays the tutorial is significantly heavy. As a result, some browsers do not render it properly and take some time to put each element in its place. In order to view the tutorial, as well as the complete workflow of DEVEA itself, we strongly recommend the use of Mozilla Firefox Web Browser. This part has been clarified in the ‘Operation’ section.**

****

*- In the example data link: https://github.com/MiriamRiquelmeP/DEVEA/tree/main/data, it would be better to include a readme file with descriptions of the example datasets.*

**+ A README.md file has been included on GitHub data folder, with all the test data inside (for Mus musculus and for Arabidopsis thaliana).**

****

*- “Supplemental-Information.txt: Running DEVEA locally on a machine”: I would recommend the author build DEVEA as an R package. Then, instead of “Download the compressed folder from the DEVEA GitHub repository”, users can install and run DEVEA on a local machine as an R package.*

**+ In fact, DEVEA is a web interface with two R Shiny applications bundled together and due to R packages structural constraints, it is not so straightforward to package such an application. However, to facilitate the local installation of DEVEA, we have added a script on DEVEA’s github that will install all the necessary R dependences for DEVEA (script_libraryApp.R). Then the user only has to clone the DEVEA repository from github and launch the application (2-steps process) after running the script. For future versions, we plan to develop a Docker image, rather than an R package. We have detailed the process in the supplemental information (https://github.com/MiriamRiquelmeP/DEVEA/blob/main/Supplemental-Information.txt)**

*- Page 3: “Despite the great progress to facilitate transcriptomics computational analyses, some improvements are still possible for these tools.” Although comparisons were given in the Related work section, the author should give brief descriptions of these tools and summarise their limitations as literature reviews in the introduction. The author also should address what are the possible improvements and why they are needed.*

**+ We have added a short paragraph in the introduction summarizing the features and limitations of the tools comparable to DEVEA in the introduction.**

****

*- Page 11: “Reads were aligned on the mouse genome (ENSEMBL GRCm38 release 96) and a count matrix was generated.” For reproducibility, the tool for read mapping and count matrix generation should be referred to and cited.*

**+ The software tools we used for this task and further details are now indicated in the main text.**

****

*-  Figure 7: the font size of labels and some details of the figures are too small. Would it be better to change the multiple plot layout to 3 rows x 2 columns?*

**+ We have tried to reformat the figure layout to 3 rows x 2 columns, but the result was really not satisfying, so we preferred to keep the original layout.**

*The paper has quite a few typos. The author should carefully check the entire manuscript again. Here, I have picked out some of them:*

*Page 3: “key steps of a complete RNA-seq analyses pipeline” should be “key steps of a complete RNA-seq analysis pipeline”*

**

*Page 3: “detailed bellow in the circular notes” should be “detailed below in the circular notes”.*

**

*Page 5: “the simple gene list (GL) can be build from” should be “the simple gene list (GL) can be built from”*

**

*Page 6: “the program extract the conditions and  calculates” should be “the program extracts the conditions and  calculates”*

**

*Page 11: “To demonstrate the usefulness of DEVEA, a RNA-seq” should be “To demonstrate the usefulness of DEVEA, an RNA-seq”*

**

*Page 12: “which offers significantly less options since” should be “which offers significantly fewer options since”*

**+ We thank the reviewer for pointing out those typos. All of them have been corrected. We have also carefully proofread the manuscript for other remaining typos.**

# Peer review: Reviewer response for version 1

**DOI:** 10.5256/f1000research.135003.r142536

**Published:** 2022-08-16

**Authors:** Hélène Hirbec

**Affiliations:** 1 Institut de Génomique Fonctionnelle, CNRS, INSERM, Université de Montpellier, Montpellier, France

In the present manuscript Riquelme-Perez and coll. are presenting the DEVEA software aimed at helping biologists with no or poor programming skills to analyse their Omics data (mainly RNA-seq). The rationale for the development of a new software is clearly explained, additionally in the manuscript, the authors compare their software to other web-based software and highlight that DEVEA is higher flexibility (see Table 1). The software is available as a web-based application but can also be installed locally.

 The description of the software is technically sound. As a matter of facts, it is based on the several well-known and highly used R packages aimed at analysing RNA-seq data. Interestingly, differential expression analysis (DEA) is performed using DESeq2 package on the most reliable tool for DEA. DEVEA is also flexible as it accepts input data from different format and at different stage of the analysis. Thus, DEVEA can be used for initial analysis of the data, but can be used at later stage of the data analysis. One very interesting feature of the DEVEA software is that it enables loading a specific background universe.

 The manuscript is clear and very well written. There is sufficient information provided in the manuscript to run the analyses proposed. However, it will be useful if the authors could provide (as supp material?) a step by step protocol for the different type of analysis (CM+SI, DO, GK+SV; GL).

 Overall, the manuscript presents a new software that appear more flexible and present several improvements compared to other similar software developed recently. DEVEA will be useful to biologists with poor programming skills to explore their omics data, in particular RNA-seq data. Although, the software is already usable, I noticed a couple of minor issues that would deserve improvements.

 Minor points that would need improvements:

- Could the authors specify if already normalized counts can be used as input in CM+SI type of analysis?
- Could the authors specify whether the software support both “.” and “,” as decimal separators;
- When uploading the GL with gene symbols, these latter are automatically converted to Ensembl identifiers, but some symbol are not recognized. It is unclear which genes are considered for EA analyses: all genes with symbol, only genes with an Ensembl identifier, only annotated genes with an Ensemble identifier?
- In some of the EA graphs, deregulated pathways/gene sets are referred to by their url rather than by the pathway name. This complicates the reading of the graphs;
- Demo data are downloadable, but the link given in the manuscript does not work;
- Downloads of the SVG images don’t seem to work, except for Chordplot in KEEG Enrichment for which the download is different. However, in this latter case, only the plot (but not the legend) is downloaded;
- The inclusion of GSEA type results in the analysis is interesting. Including the GSEA leading edge analysis in DEVEA would be an interesting add.

Further improvements that would be interesting to make:

- Enrichment analysis: In addition to GO and KEEG analyses, it would also be interesting to add other databases, such as Wiki Pathways for example.
- It will also be interesting to enable evaluating over-representation for custom genesets (i.e. specific molecular signatures of interest in the research field).

Remarks: Assessment as to whether sufficient details of the code, methods and analysis is provided to allow replication of the software development and its use by others, is beyond the competence of the reviewer.

Are the conclusions about the tool and its performance adequately supported by the findings presented in the article?

Yes

Is the rationale for developing the new software tool clearly explained?

Yes

Is the description of the software tool technically sound?

Yes

Are sufficient details of the code, methods and analysis (if applicable) provided to allow replication of the software development and its use by others?

Yes

Is sufficient information provided to allow interpretation of the expected output datasets and any results generated using the tool?

Yes

Reviewer Expertise:

Transcriptomics; Neuroinflammation; Alzheimer Disease

I confirm that I have read this submission and believe that I have an appropriate level of expertise to confirm that it is of an acceptable scientific standard.

# Response

**Published:** 2023-02-20

**Authors:** Miriam Riquelme-Perez

**Affiliations:** MIRCen (CEA - Univ. Paris-Saclay, FR), France

**Thank you for taking the time to review our paper, and for the thoughtful suggestions. In the revised version, we have modified the main text to refer more clearly to the method information provided on the interface, and improved the clarity of the text in several places, according to your suggestions. In the same way, we have included a few enhancements in the application tool itself.**

**Below are the questions raised by the reviewer (- in italic), followed by the authors' response (+ in bold):**

*- The manuscript is clear and very well written. There is sufficient information provided in the manuscript to run the analyses proposed. However, it will be useful if the authors could provide (as supp material?) a step by step protocol for the different type of analysis (CM+SI, DO, GK+SV; GL).*

**+ In addition to the article and the information within DEVEA, it was also possible to consult a comprehensive walkthrough on how to use the application from the different input modes. It was accessible from both DEVEA modules (DESeq DEVEA and Simple DEVEA) in the ‘Tutorial’ section from the top right controls inside the application interface and independently on https://shiny.imib.es/DESeqDevea/tutorial.html or https://shiny.imib.es/simpleDevea/tutorial.html. The tutorial was mentioned in the ‘Software availability’ section, and now this is mentioned in the ‘Operation’ section.**

*- Could the authors specify if already normalized counts can be used as input in CM+SI type of analysis?*

**+ The counting matrix (CM) and the sample information (SI) elements will be used as raw data to be internally analyzed through the various functions of the DESeq2 package. To build the final DESeqDataSet object, DEVEA uses the data as obtained from the counting process, presented in the form of an array of integer values. Decimals will not be accepted. The DESeq2 model internally corrects for library size and generates its own normalization, so transformed or normalized values must not be used in the CM + SI input type (as detailed in the official bioconductor DESeq2 vignette section "Why un-normalized counts?" http://bioconductor.org/packages/devel/bioc/vignettes/DESeq2/inst/doc/DESeq2.html#why-un-normalized-counts). A sentence that specifies this point is now included in the ‘Data requirement’ section, 1. CM + SI mode.**

*- Could the authors specify whether the software support both “.” and “,” as decimal separators?*

**+ Thank you for raising this point. Decimals must be indicated with "." as a separator. "," will not be properly recognized by the program.**

*- When uploading the GL with gene symbols, these latter are automatically converted to Ensembl identifiers, but some symbol are not recognized. It is unclear which genes are considered for EA analyses: all genes with symbol, only genes with an Ensembl identifier, only annotated genes with an Ensemble identifier?*

**+ The KEGG, GO, and GSEA libraries used in DEVEA need as input a list of genes coded in their ENTREZ ID format. For this reason, an exhaustive process to convert the initial gene names provided by the user (either in gene SYMBOL or ENSEMBL) into ENTREZ ID is conducted internally by DEVEA. Finally, the EA functions retrieve the associated annotation terms from these genes to generate the results. Therefore, only genes from the original data with an ENTREZ ID name can be linked to its annotation in the libraries.**

**Unfortunately, not all genes have curated ENTREZ ID names linked with the different formats or have annotated functions associated with the libraries. As a consequence, some genes will be left out of the EA. The number of genes considered in the following steps is indicated in green below the “preview table” as shown in Figure 5B. This is a limitation of the gene name conversion functions without manual curation. This point was discussed in the ‘Enrichment analysis (EA) and visualization’ subsection in the ‘Implementation’ part.**

*- In some of the EA graphs, deregulated pathways/gene sets are referred to by their url rather than by the pathway name. This complicates the reading of the graphs;*

**+ We agree that using only the URL is cumbersome since it is necessary to go back to the table to link with the actual name. Due to space limitations in some plots, pathways or functions that have a very long name cannot be shown in full. However, in most of the images where the full name cannot be displayed, it will be shown in full by placing the mouse arrowhead on top of the interactive graph.**

*- Demo data are downloadable, but the link given in the manuscript does not work;*

**+ We apologize for this problem. The link has been corrected in the manuscript and now redirects to the appropriate page to access the demo data on GitHub.**

*- Downloads of the SVG images don’t seem to work, except for Chordplot in KEGG Enrichment for which the download is different. However, in this latter case, only the plot (but not the legend) is downloaded;*

**+ Indeed, there was a server issue that has now been fixed. The functionality to export SVG images is fully operational again. Some graphics may change slightly when downloaded because the functions used to render/download are not necessarily the same as those used to display them. The plot information must remain the same.**

*- The inclusion of GSEA type results in the analysis is interesting. Including the GSEA leading edge analysis in DEVEA would be an interesting add.*

**+ We agree that the inclusion of the leading edge for GSEA results is interesting. This has been included in DEVEA as an extra column in the table at the GSEA tab, called “leading_edge”. It has also been implemented in the plot as a red line indicating the extent of what we consider the leading edge genes. This is also mentioned in the text ‘Enrichment analysis (EA) and visualization’ section.**

*- Further improvements that would be interesting to make :*

- *Enrichment analysis: In addition to GO and KEEG analyses, it would also be interesting to add other databases, such as Wiki Pathways for example.*
- *It will also be interesting to enable evaluating over-representation for custom genesets (i.e. specific molecular signatures of interest in the research field).*

**+ Regarding the first further improvement, adding more databases would be a significant extension to the current version of DEVEA, that we want to keep fast and operational. For the second suggestion, the specific custom genesets needed for the over-representation analysis could be very different between users and experiments, hence we prefer to only include the curated well known genesets from the databases. Nevertheless, DEVEA allows the definition of background genes for comparison in the EA part, avoiding the inappropriate inflation of statistical p-values and false-positive results from contrast against the whole genome/proteome.**

**For the moment, we aim at offering a stable and robust version and creating a docker container with these basic functionalities efficiently working in the server. We think that including these significant improvements is beyond the scope of this first DEVEA version. However, these are exciting suggestions to be considered in the following versions. Our goal is to further develop DEVEA according to the needs of the community and bio-informatics developments. This is acknowledged in the ‘Conclusion and Future Perspectives’ section, and we will consider including new functionalities in a future version of DEVEA.**
